# Effects of Salt-Reduction Health Education Short Videos on Salt-Related Knowledge, Attitudes, and Practices (KAP) Among Caregivers of Preschool Children

**DOI:** 10.3390/nu18213525

**Published:** 2026-10-26

**Authors:** Zhenzhen Peng, Yingjiao Deng, Du Zhang, Yaocheng Luo, Ya Shi, Huaping Wang, Zumin Shi, Yong Zhao

**Affiliations:** 1 School of Public Health, Chongqing Medical University, Chongqing 400016, China; 2025121908@stu.cqmu.edu.cn (Z.P.); 2023111615@stu.cqmu.edu.cn (Y.D.); 2024111689@stu.cqmu.edu.cn (Y.L.); 15700539291@163.com (Y.S.); 2025410054@stu.cqmu.edu.cn (H.W.); 2 Research Center for Medicine and Social Development, Chongqing Medical University, Chongqing 400016, China; 3 Research Center for Public Health Security, Chongqing Medical University, Chongqing 400016, China; 4 Nutrition Innovation Platform-Sichuan and Chongqing, School of Public Health, Chongqing Medical University, Chongqing 400016, China; 5 Human Nutrition Department, College of Health Sciences, Qatar University, Doha 2713, Qatar; zumin@qu.edu.qa

**Keywords:** health education videos, salt reduction, KAP, caregivers, preschool children

## Abstract

Background: Excessive salt intake is a major modifiable risk factor for hypertension and cardiovascular diseases. Caregivers of preschool children play an important role in shaping children’s dietary behaviors. This study aimed to evaluate the effectiveness of salt-reduction health education short videos in improving salt-related knowledge, attitudes, and practices (KAP) among caregivers of preschool children. Methods: This study enrolled 1038 caregivers of preschool children who were categorized into a short-video intervention group, a print media group, and a control group. The short-video and print media groups received a one-week salt-reduction health education intervention, whereas the control group received no additional intervention. KAP scores were assessed at pre-intervention, 1 week post-intervention, and 2 months post-intervention. Linear mixed-effects models, multivariable linear regression, and subgroup analyses were conducted to evaluate intervention effects. Results: In the linear mixed-effects model analysis, the short-video intervention group showed significantly greater increases in total KAP scores at 1 week post-intervention (β = 5.87, 95% CI: 4.30–7.43, *p* < 0.001) and 2 months post-intervention (β = 4.03, 95% CI: 2.46–5.60, *p* < 0.001). In the fully adjusted multivariable linear regression analysis, the short-video intervention group had significantly higher total KAP scores than the control group at 1 week post-intervention (β = 7.25, 95% CI: 5.66–8.84, *p* < 0.001) and 2 months post-intervention (β = 5.26, 95% CI: 3.56–6.97, *p* < 0.001). Subgroup analyses revealed that the short-video intervention group had significantly higher total KAP scores and domain-specific scores in most subgroups at 1 week and 2 months post-intervention. Conclusions: Salt-reduction health education short videos showed greater improvements in salt-related KAP among caregivers of preschool children than the print media intervention, although these differences cannot be attributed solely to the information delivery format, and short videos remain a promising strategy for salt-reduction education.

## 1. Introduction

A high-salt diet has become an important public health issue worldwide. High salt intake is not only a major determinant of elevated blood pressure (BP) and the development of hypertension, but is also associated with the risk of various cardiovascular diseases [1,2]. Studies have shown that high salt intake is significantly associated with the prevalence of hypertension among children [3,4]. The 2017 Global Burden of Disease study identified high sodium intake, low whole-grain intake, and low fruit intake as the three leading dietary risk factors worldwide [5]. The World Health Organization (WHO) recommends a daily salt intake of no more than 5 g (approximately 2 g of sodium) [6]. However, the average daily salt intake in most countries exceeds this recommended level [7,8,9,10]. Although daily salt consumption varies across provinces in China, it remains above the WHO recommendation in all regions [11]. According to the latest report, the average daily cooking salt consumption among Chinese residents decreased from 9.3 g in 2019 to 7.8 g in 2025 [12], yet remains above the maximum intake recommended by WHO.

Preschool childhood is an important period for establishing low-salt dietary habits and a critical stage for the development of dietary behaviors and lifestyles [13,14]. As the primary dietary caregivers of preschool children, caregivers’ salt-related knowledge, attitudes toward salt reduction, and salt-related practices in daily cooking and child feeding not only directly influence children’s daily salt intake, but also play a crucial role in the development of low-salt dietary habits among children [15,16,17]. A previous study showed that for every 1 g increase in a mother’s daily salt intake, the salt intake of her 3-year-old child increased by approximately 0.14 g [18]. Therefore, implementing salt-reduction interventions among caregivers of preschool children may effectively reduce excessive salt intake among children.

Knowledge, attitudes, and practices play a key role in individual behavior change by enhancing relevant knowledge and shaping beliefs and attitudes to promote the formation and maintenance of healthy behaviors [19]. Optimizing salt-reduction health education and exploring new strategies to improve health communication have increasingly attracted attention from professionals both domestically and internationally [20,21]. Traditional salt-reduction health education has primarily relied on print media materials or health lectures, but it has certain limitations in terms of coverage, participation, and sustainability [22]. In recent years, with the rapid development of the Internet, short videos have emerged as a novel form of health education, and their intuitive, vivid, easily disseminated, and engaging characteristics have gradually made them an important medium for health promotion [23]. Previous studies have shown that short videos can significantly enhance the audience’s health knowledge and facilitate behavior change [24,25].

However, research on short-video interventions targeting caregivers of preschool children remains relatively limited. This study aimed to compare the effects of salt-reduction health education short videos and print media materials on salt-related knowledge, attitudes, and practices among caregivers of preschool children. The sustained effects of the interventions at 1 week and 2 months post-intervention were also assessed, with the aim of providing evidence and practical guidance for improving dietary behaviors among preschool children.

## 2. Materials and Methods

### 2.1. Study Participants and Population

The study was conducted from April to October 2023 in 17 privately operated kindergartens of similar size across six main urban districts of Chongqing (Dadukou, Jiulongpo, Shapingba, Yubei, Jiangbei, and Beibei). With the assistance of kindergarten principals and class teachers, caregivers of preschool children were recruited as study participants for a salt-reduction health education intervention. Among the 17 eligible kindergartens, six were selected based on kindergarten size, basic characteristics, and the feasibility of study implementation, and were matched to establish three comparable study groups, with two kindergartens assigned to each group. This study employed a non-randomized controlled intervention design. Based on the kindergarten matching process and intervention arrangements, participants were categorized into three groups: a short-video intervention group, a print media group, and a control group. The short-video intervention group received self-developed salt-reduction health education short videos, the print media group received print media materials covering similar salt-reduction health education content, and the control group received no additional intervention. This study strictly adhered to the Declaration of Helsinki and was approved by the Ethics Committee of Chongqing Medical University. All participating caregivers were fully informed of the study objectives and procedures and provided written informed consent.

The inclusion and exclusion criteria for this study were as follows. Inclusion criteria: (1) being the primary caregiver of a preschool child and assuming primary responsibility for feeding; (2) spending the most time with the preschool child; (3) possessing the ability to use smart mobile devices, such as smartphones or tablet computers; (4) voluntarily agreeing to participate in the study and providing written informed consent. Exclusion criteria: (1) being unable to assume primary responsibility for the preschool child’s daily meal preparation and nutritional management; (2) having previously participated in a systematic salt-reduction intervention program; (3) having cognitive impairment or communication disorders that could affect participation in the study; (4) refusing to provide written informed consent.

**Figure 1:**
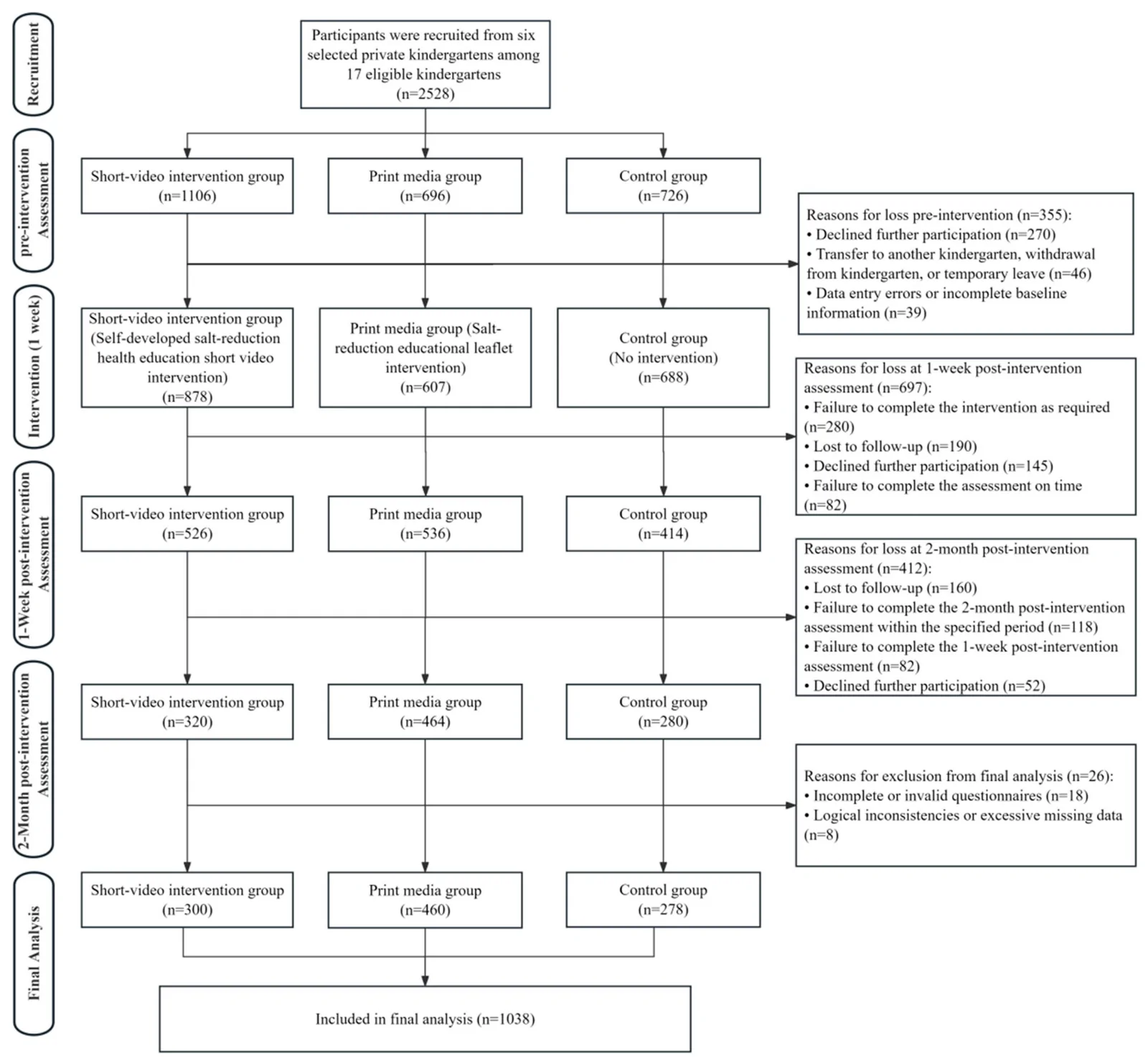
Flow chart of participant selection.

Based on previous studies, the standard deviation (σ) of salt-related KAP scores among Chinese residents was 9.18 points [26]. Assuming a two-sided significance level (α) of 0.05, a statistical power of 90%, and an expected difference of 3 points in the total KAP score between the intervention and control groups, the sample size was calculated using the formula for comparing the means of two independent samples. The results indicated that at least 197 participants were required in each group. After accounting for an anticipated loss to follow-up rate of 15%, a minimum of 232 participants was required per group. This study included three groups: a short-video intervention group, a print media group, and a control group; therefore, the total sample size required was at least 696 participants. A total of 1038 participants were ultimately included in the final analysis, which exceeded the minimum sample size requirement. The participant selection process is presented in Figure 1.

### 2.2. Intervention Materials and Methods

The intervention materials were developed based on the KAP theory and the Transtheoretical Model (TTM) [27,28], in combination with the Ten Core Messages on Salt Reduction for Chinese Residents issued by the National Health Commission of the People’s Republic of China [29]. Four short educational videos on salt reduction were developed. The video content covered: (1) the health risks associated with high salt intake and the benefits of salt reduction; (2) the recommended daily salt intake for different age groups; (3) identification of “hidden salt” in daily life (e.g., snacks, condiments, and processed foods); (4) practical salt-reduction strategies, such as using salt-restriction tools, substituting salt with natural herbs and spices, and learning to read nutrition labels. Each video lasted 1-2 min and was produced under the guidance of physicians from tertiary public hospitals and experts in food and nutritional sciences, ensuring that the content was scientifically accurate and easy to understand.

The print media materials presented salt-reduction health education content using a combination of text and illustrations and were compiled and printed as paper-based salt-reduction educational leaflets. The main educational content was similar to that of the salt-reduction health education short videos.

Within one week after the baseline survey, a short health education video on salt reduction was delivered every other day to caregivers via WeChat groups (Tencent, Shenzhen, Guangdong, China) established for each kindergarten class (which function similarly to WhatsApp groups). Four videos were delivered during the intervention week: on Monday, “Health Risks of Salt and Benefits of Reduction”; on Wednesday, “Recommended Daily Salt Intake for Different Age Groups”; on Friday, “Identifying Hidden Salt”; and on Sunday, “Practical Salt-Reduction Tips”. For caregivers in the print media group, salt-reduction print educational materials were distributed after the baseline survey during caregiver meetings or kindergarten entry activities organized by class teachers.

### 2.3. Questionnaire Design

A self-developed questionnaire, the “Survey on Salt Intake Status and Salt-Reduction Knowledge, Attitudes, and Practices among Caregivers of Preschool Children”, was administered in this study, and its reliability and validity were assessed. The reliability and validity analyses showed a Cronbach’s α coefficient of 0.711, a KMO value of 0.7, and a significant Bartlett’s test of sphericity (*p* < 0.05), indicating acceptable reliability and validity. The questionnaire consisted of 53 items across four main sections: (1) caregiver and preschool child characteristics (17 items, including caregiver sex, age, educational level, average monthly household income level, and other demographic characteristics); (2) salt-reduction knowledge (9 items covering the health risks of excessive salt intake and recommended salt intake levels); (3) salt-reduction attitudes (7 items assessed using a five-point Likert scale); and (4) salt-reduction practices (20 items addressing household cooking salt use, salt intake from snacks, and consumption of foods containing “hidden salt”).

### 2.4. Outcome Variables

The self-developed questionnaire was administered through Questionnaire Star (Changsha Ranxing Information Technology Co., Ltd., Changsha, Hunan, China), an online survey platform, and distributed by teachers to caregivers via WeChat groups. The effects of the intervention were evaluated at three time points: pre-intervention (baseline), 1 week post-intervention, and 2 months post-intervention. The questionnaire was scored across three domains: salt-reduction knowledge (total score: 0–10), salt-reduction attitudes (total score: 0–24), and salt-reduction practices (total score: 0–60), yielding a total KAP score ranging from 0 to 96. Scores for knowledge, attitudes, practices, and total KAP were converted into percentage scores using a relative scoring method, with higher scores indicating better salt-reduction knowledge, more positive attitudes, and healthier practices.

The post-intervention questionnaires were consistent with the baseline questionnaire in terms of content and scoring methodology, except that demographic information collected at baseline was not repeated.

### 2.5. Data Quality Control

All researchers and class teachers received standardized training to ensure a comprehensive understanding of the study rationale, objectives, and procedures. In addition, a feedback system was established. Researchers monitored data quality and study progress in real time via Questionnaire Star and maintained communication with class teachers to promptly coordinate and resolve issues, thereby ensuring the completeness and validity of data collection. Finally, all collected questionnaires were carefully reviewed, and invalid questionnaires were excluded after discussion.

### 2.6. Data Processing and Statistical Analyses

Data analyses were performed using Stata 18.0 (StataCorp LLC, College Station, TX, USA). Continuous variables were expressed as mean ± standard deviation (M ± SD), and categorical variables as frequencies and percentages (*n*, %). Baseline characteristics were compared using the chi-square test or one-way ANOVA. Children aged 3 and 4 years were classified into the 3–4-year group, while those aged 5 and 6 years were classified into the 5–6-year group. The BMI classifications of caregivers and preschool children were determined according to the “Guidelines for Weight Management (2024 Edition)” issued by the National Health Commission of the People’s Republic of China [30]. A linear mixed-effects model was used to assess changes in KAP total and domain scores over time. Group (control, print media, short video) was included as a between-subjects factor, and time (pre-intervention, 1 week post-intervention, 2 months post-intervention) as a within-subject factor. The control group and pre-intervention were set as references, and participant ID was included as a random effect to account for within-subject correlations. Main effects of group, time, and their interaction were tested. Multivariable linear regression analyses were further conducted to examine the association between intervention groups and KAP scores at 1 week and 2 months, using the control group as the reference. Three models were constructed: Model 1 unadjusted; Model 2 adjusted for caregiver sex and age; Model 3 additionally adjusted for BMI, education level, household income, occupation, family disease history, number of children, caregiver–child relationship, and child characteristics. Subgroup analyses were performed to explore the consistency of intervention effects across different populations, and interaction terms between subgroup variables and intervention groups were tested. All tests were two-sided, and *p* < 0.05 was considered statistically significant.

## 3. Results

### 3.1. Basic Demographic Features

A total of 1038 caregivers of preschool children were included in this study. Of these, 78.9% were female, with a mean age of 34.07 ± 5.94 years. Most caregivers were mothers (84.1%), and 66.5% had a university education or higher. Regarding household income, 56.7% reported a monthly income of ≥10,000 RMB. Caregivers’ BMI was predominantly within the normal range (64.0%), and approximately 66.1% of households had at least one family member with a chronic disease. Detailed characteristics are presented in Table 1.

### 3.2. Descriptive Analysis

Table 2 shows that the short-video intervention group showed a relatively marked increasing trend in total KAP scores, with scores increasing from 64.13 ± 9.24 at pre-intervention to 71.70 ± 10.12 at 1 week post-intervention and remaining at 70.91 ± 10.57 at 2 months post-intervention. In contrast, the changes in total KAP scores in the print media group and the control group were relatively smaller, with scores of 66.33 ± 10.72 and 65.80 ± 10.46, respectively, at 2 months post-intervention. Among the KAP domains, knowledge scores showed the greatest change in the short-video intervention group, increasing from 37.40 ± 22.46 at pre-intervention to 72.83 ± 22.52 at 1 week post-intervention. Although the knowledge scores decreased at 2 months post-intervention (67.27 ± 24.25), they remained higher than the pre-intervention level. In contrast, attitude and practice scores showed relatively smaller changes across groups.

### 3.3. Linear Mixed-Effects Model Analysis

Table 3 shows that the main effect of group was not significant for total KAP scores or any domain-specific scores (all *p* > 0.05). Regarding the main effect of time, total KAP scores significantly increased at 1 week post-intervention (β = 1.70, 95% CI: 0.57–2.82, *p* = 0.003) and 2 months post-intervention (β = 2.75, 95% CI: 1.62–3.88, *p* < 0.001) compared with pre-intervention. Similarly, knowledge scores were significantly higher at 1 week (β = 7.01, 95% CI: 4.32–9.71, *p* < 0.001) and 2 months post-intervention (β = 13.27, 95% CI: 10.58–15.97, *p* < 0.001), and practice scores showed significant increases at 1 week (β = 1.67, 95% CI: 0.50–2.83, *p* = 0.005) and 2 months post-intervention (β = 3.21, 95% CI: 2.04–4.38, *p* < 0.001). However, no significant change in attitude scores was observed at 1 week post-intervention (*p* = 0.735), while attitude scores were significantly lower at 2 months post-intervention (β = −2.77, 95% CI: −5.38-−0.17, *p* = 0.037).

Analysis of the group-by-time interaction revealed that, compared with the control group, the short-video intervention group showed significantly greater increases in total KAP scores at 1 week (β = 5.87, 95% CI: 4.30–7.43, *p* < 0.001) and 2 months post-intervention (β = 4.03, 95% CI: 2.46–5.60, *p* < 0.001). The short-video intervention group also demonstrated substantial improvements in knowledge scores at 1 week (β = 28.42, 95% CI: 24.68–32.16, *p* < 0.001) and 2 months post-intervention (β = 16.59, 95% CI: 12.85–20.34, *p* < 0.001). In addition, significantly higher attitude scores were observed in the short-video intervention group at both 1 week (β = 4.84, 95% CI: 1.23–8.45, *p* = 0.009) and 2 months post-intervention (β = 5.18, 95% CI: 1.56–8.79, *p* = 0.005). For practice scores, the short-video intervention group showed a significant improvement at 1 week post-intervention (β = 2.52, 95% CI: 0.90–4.14, *p* = 0.002), whereas the difference was not statistically significant at 2 months post-intervention (*p* = 0.074). In contrast, the print media group showed a significant increase only in total KAP scores at 1 week post-intervention (β = 1.55, 95% CI: 0.12–2.98, *p* = 0.034), with no significant differences observed for other outcomes or time points (all *p* > 0.05).

**Figure 2:**
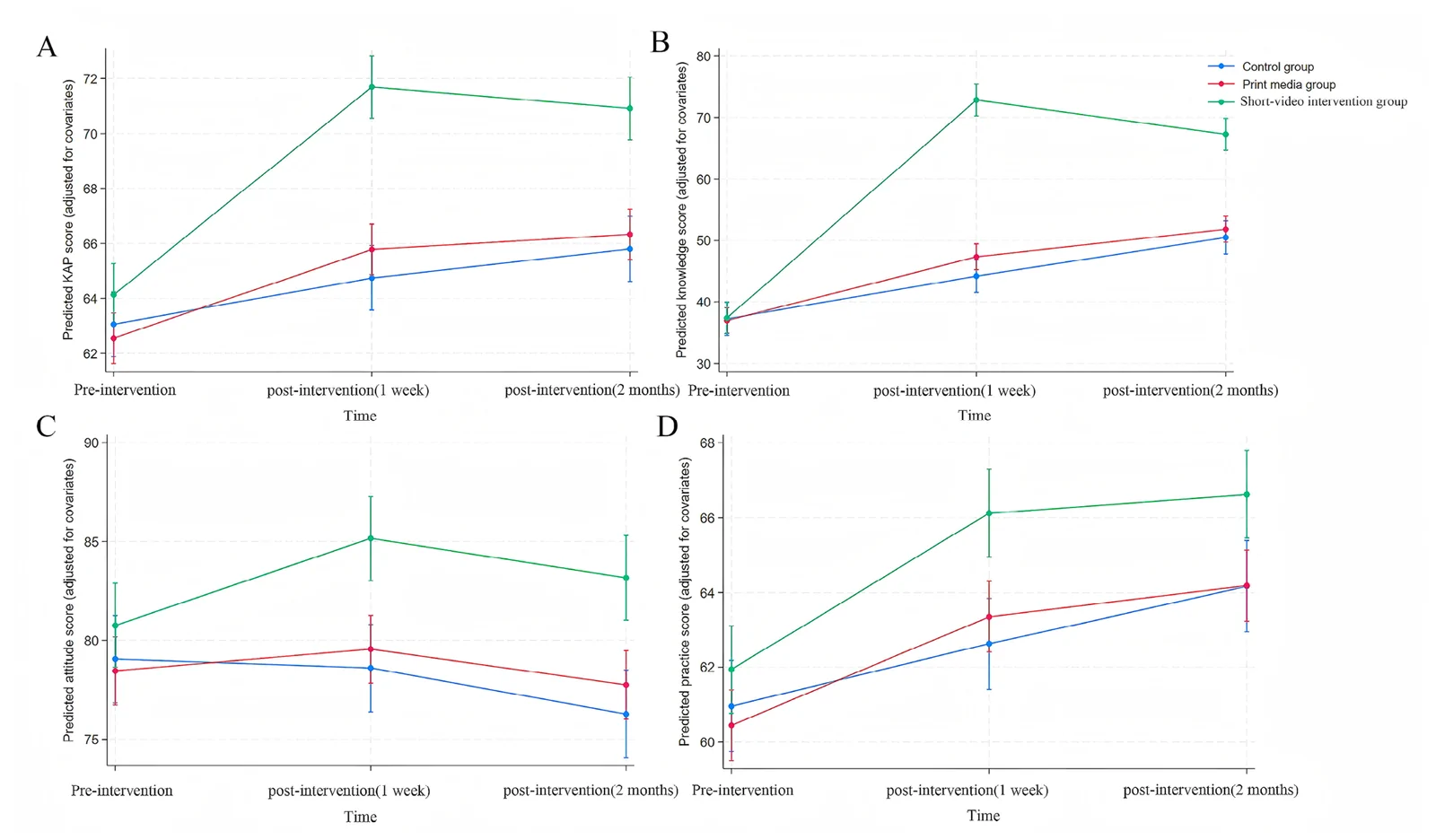
Adjusted mean scores of total KAP and domain-specific scores of caregivers of preschool children across groups and time points. (**A**) Adjusted mean total KAP scores across groups. (**B**) Adjusted mean knowledge scores across groups. (**C**) Adjusted mean attitude scores across groups. (**D**) Adjusted mean practice scores across groups. Blue: control group; red: print media group; green: short-video intervention group. Values are means and 95% confidence intervals.

Figure 2 presents the adjusted mean scores of total KAP, knowledge, attitude, and practice among caregivers of preschool children across groups and time points.

### 3.4. Multivariable Linear Regression Analysis

Table 4 shows that at 1 week post-intervention, the short-video intervention group had significantly higher total KAP scores and domain-specific scores than the control group in the fully adjusted model (Model 3), including total KAP scores (β = 7.25, 95% CI: 5.66–8.84, *p* < 0.001), knowledge scores (β = 29.20, 95% CI: 25.64–32.77, *p* < 0.001), attitude scores (β = 6.85, 95% CI: 3.75–9.96, *p* < 0.001), and practice scores (β = 3.75, 95% CI: 2.06–5.44, *p* < 0.001). In contrast, the print media group showed a significant association only with knowledge scores in Model 3 (β = 3.30, 95% CI: 0.01–6.60, *p* = 0.049).

Table 5 shows that at 2 months post-intervention, the short-video intervention group exhibited significantly higher total KAP scores and domain-specific scores than the control group in Model 3, including total KAP scores (β = 5.26, 95% CI: 3.56–6.97, *p* < 0.001), knowledge scores (β = 17.07, 95% CI: 13.28–20.86, *p* < 0.001), attitude scores (β = 7.08, 95% CI: 3.85–10.31, *p* < 0.001), and practice scores (β = 2.57, 95% CI: 0.85–4.29, *p* = 0.004). However, no significant associations were observed between the print media group and total KAP scores or any domain-specific scores (all *p* > 0.05).

### 3.5. Subgroup Analysis

The results showed that at 1 week post-intervention, the short video intervention group showed significantly higher total KAP scores and all domain-specific scores in most subgroups, with the largest improvement observed in the knowledge domain. In contrast, the print media group showed weaker effects across most subgroups, with no significant differences. Interaction analysis indicated that only occupation was significantly associated with the group effect on total KAP scores (*p* for interaction = 0.013), whereas no significant interactions were observed for other subgroup factors (all *p* for interaction > 0.05).

At 2 months post-intervention, the short video intervention group continued to show significant improvements in total KAP scores and all domain-specific scores in most subgroups, with the knowledge domain remaining the most improved, although the overall effect was slightly reduced compared with 1 week post-intervention. The print media group showed modest effects in a few subgroups, but most subgroup differences were not statistically significant. Interaction analyses revealed no significant interactions between subgroup factors and intervention groups (all *p* for interaction > 0.05). Detailed results are presented in Supplementary Tables S1–S8.

## 4. Discussion

In this study, caregivers of preschool children received a 1-week salt-reduction intervention delivered through either self-developed salt-reduction health education short videos or print media materials. The intervention effects were evaluated at 1 week and 2 months post-intervention. By comparing total KAP scores and domain-specific scores related to salt intake among caregivers across different time points and intervention modalities, this study assessed the effectiveness and sustainability of short-video-based health education in promoting salt reduction among caregivers.

This study found that among the 1038 caregivers included, overall salt-related knowledge was relatively low, whereas attitudes toward salt reduction were generally favorable, with practice scores falling between the two. A community-based survey conducted in Australia reported that although most participants expressed a willingness to reduce salt intake, their understanding of dietary salt sources and related health risks remained inadequate, and the addition of extra salt during daily cooking was still common [31]. Similarly, several cross-sectional studies conducted in China have shown that Chinese residents generally have insufficient salt-related knowledge and strong intentions to reduce salt intake, while actual improvements in salt reduction practices remain limited [26,32,33]. This imbalance among knowledge, attitudes, and practices suggests that individual health awareness alone may be insufficient to achieve sustained behavioral change, highlighting the importance of strengthening targeted salt reduction health education among priority populations. However, traditional print media may have limitations in terms of reader engagement and the accessibility of health information, depending on the target population and delivery context.

Further analyses using linear mixed-effects models demonstrated that the short-video intervention group showed greater improvements in total KAP scores and domain-specific scores compared with the control group, with significant group-by-time interaction effects observed for total KAP scores, knowledge scores, and attitude scores at both 1 week and 2 months post-intervention. For practice scores, the interaction effect was significant at 1 week post-intervention but was no longer statistically significant at 2 months post-intervention. Khokhar et al., based on the DELISH digital salt reduction project, reported that digital health education could significantly improve parents’ salt-related KAP levels [34], which was consistent with the findings of the present study. Compared with the print media intervention, the short-video intervention showed greater improvements in salt-related KAP outcomes in the present study. However, because the wording, illustrative materials, and exposure time were not fully standardized between the two intervention groups, these differences cannot be attributed solely to the delivery format. The findings should therefore be interpreted as evidence supporting the effectiveness of a short-video-based health education approach under the conditions of the present intervention rather than as evidence of an independent effect of the short-video format itself.

Notably, although the intervention effects remained significant at 2 months post-intervention compared with pre-intervention, total KAP scores as well as knowledge and attitude scores in the short-video intervention group decreased to varying degrees compared with 1 week post-intervention, suggesting that the effects of a one-time, short-term health education intervention may gradually attenuate over time. Similarly, a community-based randomized controlled trial on salt reduction conducted by Wang et al. reported a mild decline in participants’ KAP scores during the follow-up period after the intervention [35]. Yuan et al. demonstrated that a 12-month combined family- and school-based salt reduction intervention could significantly improve the KAP levels of both children and their parents; although the intervention effect slightly attenuated at the 24-month follow-up, overall levels remained above baseline [36]. These studies highlight that health behavior change is a long-term and dynamic process, and sustained reinforcement and repeated exposure are essential for maintaining behavioral improvements. Therefore, future interventions could explore establishing long-term dissemination mechanisms through new media channels such as WeChat public accounts and short video platforms, with regular delivery of salt reduction-related health education content to continuously reinforce health knowledge and healthy dietary practices. To facilitate sustained implementation, these short-video resources could be incorporated into routine kindergarten-based health education and parent engagement activities, rather than being provided only as a short-term intervention. In addition, standardized and evidence-based short-video materials could be developed as a reproducible health education package and disseminated through collaboration among kindergartens, primary healthcare institutions, and public health agencies. Such an approach could support the implementation of the intervention across a larger number of kindergartens and different geographic regions, thereby enhancing its scalability.

Subgroup analyses revealed that occupation was the only factor significantly associated with changes in total KAP scores at 1 week post-intervention, suggesting that occupational background may be associated with differences in intervention effects. Differences in educational attainment, health literacy, access to health information, and media use habits across occupational groups may affect individuals’ receptivity to and comprehension of health education content. Previous studies have demonstrated that occupation is closely associated with residents’ health knowledge, dietary practices, and awareness of chronic disease prevention and control [37], which is consistent with the findings of the present study. Primary family caregivers play a crucial role in shaping children’s dietary behaviors. Existing evidence has shown that family caregivers, particularly parents, exert a substantial influence on the establishment of children’s dietary patterns, food choices, and health behaviors [38,39]. Therefore, future salt reduction health education interventions should further optimize the design and dissemination strategies of short video content according to the information acquisition characteristics and caregiving patterns of different occupational groups, thereby enhancing the precision and relevance of health communication.

Reducing sodium intake is considered one of the most cost-effective public health strategies and plays a crucial role in lowering the burden of hypertension and other non-communicable diseases [40,41]. In recent years, various salt reduction measures have been implemented both domestically and internationally, including the promotion of low-sodium salt, reformulation of food products, optimization of food labeling, and interventions targeting the food environment [42,43,44]. In China, policies such as the Healthy China Action (2019–2030) have continuously promoted population-level salt reduction [45]. However, existing studies indicate that public awareness of recommended salt intake, the health risks of high-salt diets, and hidden sources of dietary sodium remains relatively low [46,47]. Many households continue to add excessive salt during daily cooking and seasoning, suggesting that policy advocacy and traditional educational approaches alone are insufficient to achieve widespread and sustained behavioral change. The findings of the present study indicate that short videos, as an emerging form of health communication, can effectively enhance caregivers’ knowledge and salt reduction practices, providing a novel approach for salt reduction education among children and offering a reference for future digital health promotion initiatives.

This study has several strengths. First, this study included three comparison groups receiving different health education approaches, including a short-video intervention group, a print media group, and a control group. The comparison of changes in salt-related KAP outcomes before and after the intervention allowed the effectiveness of the different health education approaches to be evaluated under the conditions of the present study. Second, this study targeted caregivers of preschool children, who play an important role in making and implementing children’s dietary decisions. Improving caregivers’ salt-related knowledge, attitudes, and practices may contribute to a healthier dietary environment for preschool children and has important implications for family-based dietary health promotion. Third, linear mixed-effects models were applied to analyze repeated-measures data, accounting for correlations among measurements from the same participants and improving the accuracy and robustness of the estimated intervention effects. Finally, subgroup analyses were performed to explore potential differences in intervention effects across different caregiver characteristics, providing evidence for the development of more targeted salt reduction health education strategies.

This study also has several limitations. First, this was a non-randomized intervention study, and participants were not randomly assigned to different intervention groups, which may have introduced selection bias and residual confounding. Second, the participating kindergartens were selected using a convenience sampling approach and were not intended to be representative of all kindergartens in Chongqing. Their relatively concentrated geographic distribution may have introduced selection bias and limited the generalizability of the findings. Third, although the short videos and the print media materials covered similar main educational content, the exact wording, illustrative materials, and exposure time were not fully standardized between the short-video and print media interventions. Therefore, the present study evaluated the effects of two complete health education approaches in a real-world setting rather than isolating the independent causal effect of the information delivery format. The observed differences in effectiveness may therefore not be attributable solely to the mode of information delivery, as differences in other intervention characteristics may also have contributed to the findings. Future studies should use intervention materials with identical content, wording, and visual information and standardize exposure time to more rigorously evaluate the independent effect of the delivery format. Fourth, salt-related knowledge, attitudes, and practices were assessed using self-reported questionnaires, which may have been affected by recall bias and social desirability bias, potentially leading to an overestimation of improvements in salt-related practices. Fifth, this study relied mainly on KAP questionnaires and did not collect objective indicators, such as 24-h urinary sodium excretion, limiting the ability to directly evaluate changes in actual salt intake. Finally, the follow-up period was limited to 2 months, which may not have been sufficient to fully assess the long-term maintenance of behavioral changes. Future studies with randomized designs, longer follow-up periods, and multi-center recruitment are warranted to further verify the sustained effects and broader applicability of short-video-based salt reduction interventions.

## 5. Conclusions

In summary, salt-reduction health education short videos showed greater improvements in salt-related knowledge, attitudes, and practices among caregivers of preschool children than the print media intervention and the control condition under the conditions of the present study. Follow-up assessments at 1 week and 2 months post-intervention indicated that these improvements were maintained to some extent over the medium term. However, because the intervention materials were not fully standardized beyond their core knowledge content, the observed differences should not be interpreted as demonstrating an independent causal effect of the short-video format. Further studies using strictly standardized intervention content and exposure conditions are needed to determine the independent contribution of the delivery format.

## Figures and Tables

**Table 1:** Demographic characteristics of caregivers of preschool children, *n* (%).

Baseline Characteristics		Total (*n* = 1038)	Control Group (*n* = 278)	Print Media Group (*n* = 460)	Short-Video Intervention Group (*n* = 300)	*p*
Caregiver sex	Male	219 (21.1)	54 (19.4)	102 (22.2)	63 (21.0)	0.670
	Female	819 (78.9)	224 (80.6)	358 (77.8)	237 (79.0)	
Caregiver age, mean (SD)	Year	34.07 ± 5.94	33.99 ± 6.12	34.16 ± 5.17	34.01 ± 6.81	0.910
Caregiver BMI	Underweight	100 (9.6)	31 (11.2)	40 (8.7)	29 (9.7)	0.790
	Normal weight	664 (64.0)	173 (62.2)	306 (66.5)	185 (61.7)	
	Overweight	177 (17.1)	48 (17.3)	75 (16.3)	54 (18.0)	
	Obese	97 (9.3)	26 (9.4)	39 (8.5)	32 (10.7)	
Presence of chronic disease in family	Yes	686 (66.1)	187 (67.3)	300 (65.2)	199 (66.3)	0.850
	No	352 (33.9)	91 (32.7)	160 (34.8)	101 (33.7)	
Caregiver education level	Junior high school or below	99 (9.5)	32 (11.5)	34 (7.4)	33 (11.0)	0.083
	Senior high school	249 (24.0)	75 (27.0)	101 (22.0)	73 (24.3)	
	College degree or above	690 (66.5)	171 (61.5)	325 (70.7)	194 (64.7)	
Monthly household income	≤3000 RMB	65 (6.3)	12 (4.3)	32 (7.0)	21 (7.0)	0.230
	3001–5000 RMB	127 (12.2)	41 (14.7)	45 (9.8)	41 (13.7)	
	5001–10,000 RMB	257 (24.8)	72 (25.9)	109 (23.7)	76 (25.3)	
	≥ 10,000 RMB	589 (56.7)	153 (55.0)	274 (59.6)	162 (54.0)	
Caregiver occupation	Business/Service industry	253 (24.4)	75 (27.0)	113 (24.6)	65 (21.7)	0.066
	Public institution	172 (16.6)	39 (14.0)	89 (19.3)	44 (14.7)	
	Industry/Manufacturing	165 (15.9)	39 (14.0)	81 (17.6)	45 (15.0)	
	Other	448 (43.2)	125 (45.0)	177 (38.5)	146 (48.7)	
Number of children cared for	1	551 (53.1)	153 (55.0)	218 (47.4)	180 (60.0)	0.002
	≥2	487 (46.9)	125 (45.0)	242 (52.6)	120 (40.0)	
Relationship with the child	Mother–child	873 (84.1)	243 (87.4)	388 (84.3)	242 (80.7)	0.031
	Father–child	113 (10.9)	19 (6.8)	56 (12.2)	38 (12.7)	
	Other	52 (5.0)	16 (5.8)	16 (3.5)	20 (6.7)	
Child sex	Male	546 (52.6)	147 (52.9)	249 (54.1)	150 (50.0)	0.530
	Female	492 (47.4)	131 (47.1)	211 (45.9)	150 (50.0)	
Child age	3–4 years	251 (24.2)	91 (32.7)	86 (18.7)	74 (24.7)	<0.001
	5–6 years	787 (75.8)	187 (67.3)	374 (81.3)	226 (75.3)	
Child BMI	Underweight	51 (4.9)	15 (5.4)	26 (5.7)	10 (3.3)	0.480
	Normal weight	670 (64.6)	173 (62.2)	295 (64.1)	202 (67.3)	
	Overweight	113 (10.9)	27 (9.7)	55 (12.0)	31 (10.3)	
	Obese	204 (19.7)	63 (22.7)	84 (18.3)	57 (19.0)	

**Table 2:** Total and domain-specific KAP scores (mean ± SD) of caregivers of preschool children across groups and time points.

Group	Time	*n*	KAP	Knowledge	Attitude	Practice
Short-video intervention group	pre-intervention	300	64.13 ± 9.24	37.40 ± 22.46	80.76 ± 17.04	61.94 ± 9.44
	1 week post-intervention	300	71.70 ± 10.12	72.83 ± 22.52	85.15 ± 18.53	66.12 ± 11.38
	2 months post-intervention	300	70.91 ± 10.57	67.27 ± 24.25	83.17 ± 16.97	66.62 ± 10.86
Print media group	pre-intervention	460	62.54 ± 9.76	36.96 ± 22.23	78.45 ± 19.14	60.45 ± 10.10
	1 week post-intervention	460	65.79 ± 9.62	47.37 ± 23.51	79.55 ± 18.55	63.35 ± 9.87
	2 months post-intervention	460	66.33 ± 10.72	51.83 ± 22.33	77.74 ± 20.86	64.18 ± 11.12
Control group	pre-intervention	278	63.05 ± 9.82	37.23 ± 21.44	79.05 ± 17.03	60.96 ± 10.61
	1 week post-intervention	278	64.75 ± 9.84	44.24 ± 21.88	78.60 ± 19.59	62.63 ± 9.96
	2 months post-intervention	278	65.80 ± 10.46	50.50 ± 24.75	76.27 ± 20.20	64.17 ± 9.61

**Table 3:** Linear mixed-effects model analysis of total and domain-specific KAP scores of caregivers of preschool children.

	KAP		Knowledge		Attitude		Practice	
Variable	β 95% CI	*p*	β 95% CI	*p*	β 95% CI	*p*	β 95% CI	*p*
Group								
Control group	1.0 (Ref)		1.0 (Ref)		1.0 (Ref)		1.0 (Ref)	
Print media group	−0.51 (−2.00, 0.98)	0.504	−0.27 (−3.67, 3.12)	0.875	−0.60 (−3.40, 2.21)	0.677	−0.51 (−2.06, 1.03)	0.514
Short-video intervention group	1.08 (−0.55, 2.72)	0.195	0.17 (−3.55, 3.89)	0.929	1.72 (−1.36, 4.79)	0.273	0.98 (−0.71, 2.67)	0.256
Time								
pre-intervention	1.0 (Ref)		1.0 (Ref)		1.0 (Ref)		1.0 (Ref)	
1 week post-intervention	1.70 (0.57, 2.82)	0.003	7.01 (4.32, 9.71)	0.000	−0.45 (−3.05, 2.15)	0.735	1.67 (0.50, 2.83)	0.005
2 months post-intervention	2.75 (1.62, 3.88)	0.000	13.27 (10.58, 15.97)	0.000	−2.77 (−5.38, −0.17)	0.037	3.21 (2.04, 4.38)	0.000
Group × Time Interaction								
Control group × Pre-intervention	1.0 (Ref)		1.0 (Ref)		1.0 (Ref)		1.0 (Ref)	
Print media group × 1 week post-intervention	1.55 (0.12, 2.98)	0.034	3.40 (−0.02, 6.81)	0.051	1.55 (−1.75, 4.84)	0.358	1.24 (−0.24, 2.72)	0.101
Print media group × 2 months post-intervention	1.04 (−0.39, 2.47)	0.155	1.60 (−1.82, 5.01)	0.360	2.07 (−1.23, 5.36)	0.219	0.53 (−0.95, 2.01)	0.481
Short-video intervention group × 1 week post-intervention	5.87 (4.30, 7.43)	0.000	28.42 (24.68, 32.16)	0.000	4.84(1.23, 8.45)	0.009	2.52 (0.90, 4.14)	0.002
Short-video intervention group × 2 months post-intervention	4.03 (2.46, 5.60)	0.000	16.59 (12.85, 20.34)	0.000	5.18 (1.56, 8.79)	0.005	1.48 (−0.15, 3.10)	0.074

**Table 4:** Linear regression analysis of total and domain-specific KAP scores of caregivers of preschool children at 1 week post-intervention.

		KAP		Knowledge		Attitude		Practice	
		β 95% CI	*p*	β 95% CI	*p*	β 95% CI	*p*	β 95% CI	*p*
Model 1	Control group	1.0 (Ref)		1.0 (Ref)		1.0 (Ref)		1.0 (Ref)	
	Print media group	1.04 (−0.43, 2.50)	0.165	3.12 (−0.27, 6.52)	0.071	0.95 (−1.86, 3.76)	0.507	0.73 (−0.82, 2.27)	0.356
	Short-video intervention group	6.95 (5.34, 8.55)	0.000	28.59(24.86, 32.31)	0.000	6.56 (3.48, 9.63)	0.000	3.50 (1.81, 5.19)	0.000
Model 2	Control group	1.0 (Ref)		1.0 (Ref)		1.0 (Ref)		1.0 (Ref)	
	Print media group	1.01(-0.45, 2.47)	0.174	3.02 (-0.34, 6.38)	0.078	0.79 (−2.02, 3.59)	0.582	0.78 (-0.77, 2.31)	0.329
	Short-video intervention group	6.98 (5.39, 8.57)	0.000	28.69 (25.01, 32.36)	0.000	6.54 (3.48, 9.61)	0.000	3.54 (1.86, 5.22)	0.000
Model 3	Control group	1.0 (Ref)		1.0 (Ref)		1.0 (Ref)		1.0 (Ref)	
	Print media group	1.41 (−0.06, 2.88)	0.059	3.30 (0.01, 6.60)	0.049	1.34 (−1.53, 4.21)	0.360	1.13 (−0.44, 2.69)	0.158
	Short-video intervention group	7.25 (5.66, 8.84)	0.000	29.20 (25.64, 32.77)	0.000	6.85 (3.75, 9.96)	0.000	3.75 (2.06, 5.44)	0.000

Model 1 was the unadjusted model. Model 2 was additionally adjusted for caregivers’ sex and age. Model 3 was further adjusted for caregivers’ BMI, educational level, average monthly household income, occupation, family history of chronic diseases, number of children under the caregiver’s care, relationship to the child, and child-related characteristics based on Model 2.

**Table 5:** Linear regression analysis of total and domain-specific KAP scores of caregivers of preschool children at 2 months post-intervention.

		KAP		Knowledge		Attitude		Practice	
		β 95% CI	*p*	β 95% CI	*p*	β 95% CI	*p*	β 95% CI	*p*
Model 1	Control group	1.0 (Ref)		1.0 (Ref)		1.0 (Ref)		1.0 (Ref)	
	Print media group	0.53 (−1.05, 2.11)	0.513	1.32 (−2.19, 4.83)	0.460	1.47 (−1.46, 4.40)	0.324	0.02 (−1.57, 1.61)	0.982
	Short-video intervention group	5.11 (3.38, 6.84)	0.000	16.76 (12.91, 20.61)	0.000	6.89 (3.69, 10.10)	0.000	2.46 (0.71, 4.20)	0.006
Model 2	Control group	1.0 (Ref)		1.0 (Ref)		1.0 (Ref)		1.0 (Ref)	
	Print media group	0.54 (−1.04, 2.11)	0.504	1.38 (−2.14, 4.90)	0.441	1.43 (−1.51, 4.36)	0.340	0.04 (−1.54, 1.62)	0.959
	Short-video intervention group	5.14 (3.42, 6.87)	0.000	16.82 (12.97, 20.66)	0.000	6.89 (3.69, 10.10)	0.000	2.50 (0.77, 4.23)	0.005
Model 3	Control group	1.0 (Ref)		1.0 (Ref)		1.0 (Ref)		1.0 (Ref)	
	Print media group	0.69 (−0.88, 2.27)	0.387	1.13 (−2.37, 4.63)	0.527	1.90 (−1.08, 4.89)	0.211	0.14 (−1.45, 1.73)	0.865
	Short-video intervention group	5.26 (3.56, 6.97)	0.000	17.07 (13.28, 20.86)	0.000	7.08 (3.85, 10.31)	0.000	2.57 (0.85, 4.29)	0.004

Model 1 was the unadjusted model. Model 2 was additionally adjusted for caregivers’ sex and age. Model 3 was further adjusted for caregivers’ BMI, educational level, average monthly household income, occupation, family history of chronic diseases, number of children under the caregiver’s care, relationship to the child, and child-related characteristics based on Model 2.

## Data Availability

The raw data supporting the conclusions of this article will be made available by the authors, without undue reservations.

## Supplementary Materials

The following supporting information can be downloaded at https://www.mdpi.com/article/10.3390/nu18213525/s1. Table S1: Subgroup analysis of total KAP scores among caregivers of preschool children at 1 week post-intervention. Table S2: Subgroup analysis of knowledge scores among caregivers of preschool children at 1 week post-intervention. Table S3: Subgroup analysis of attitude scores among caregivers of preschool children at 1 week post-intervention. Table S4: Subgroup analysis of practice scores among caregivers of preschool children at 1 week post-intervention. Table S5: Subgroup analysis of total KAP scores among caregivers of preschool children at 2 months post-intervention. Table S6: Subgroup analysis of knowledge scores among caregivers of preschool children at 2 months post-intervention. Table S7: Subgroup analysis of attitude scores among caregivers of preschool children at 2 months post-intervention. Table S8: Subgroup analysis of practice scores among caregivers of preschool children at 2 months post-intervention. File S1: The questionnaire for this study. File S2: Comparison of core health education content between print media materials and salt-reduction health education short videos.

## Abbreviations

The following abbreviations are used in this manuscript:

KAP	knowledge, attitudes, and practices
BMI	Body mass index
