# Supplementary material for: Effects of Salt-Reduction Health Education Short Videos on Salt-Related Knowledge, Attitudes, and Practices (KAP) Among Caregivers of Preschool Children
Source: Nutrients. 2026 Oct 26;18(21):3525. doi: 10.3390/nu18213525 (PMC13764901; doi:10.3390/nu18213525)
Supplement: Supplementary file 1 [file nutrients-18-03525-s001.zip › Supplementary Materials.pdf]

Supplementary Materials

Effects of Salt-Reduction Health Education Short Videos on Salt-Related Knowledge, Attitudes, and Practices (KAP) Among Caregivers of Preschool Children

Table S1. Subgroup analysis of total KAP scores among caregivers of preschool children at 1 week post-intervention

Variable	Group			*P* for Trend	*P* for Interaction
	Control group	Print media group	Short-video intervention group		
Caregiver sex					0.063
Male	1.0 (Ref)	-0.12 (-3.36 - 3.13)	8.50 (4.87 - 12.13)	0.000	
Female	1.0 (Ref)	1.95 (0.28 - 3.62)	6.91 (5.11 - 8.71)	0.000	
Caregiver age					0.056
≤ 30 years	1.0 (Ref)	3.37 (-0.76 - 7.49)	6.10 (2.02 - 10.18)	0.004	
31-40 years	1.0 (Ref)	0.30 (-1.39 - 1.99)	7.35 (5.48 - 9.21)	0.000	
> 40 years	1.0 (Ref)	5.24 (0.51 - 9.97)	6.24 (1.25 - 11.23)	0.016	
Caregiver BMI					0.702
Underweight	1.0 (Ref)	3.82 (-1.23 - 8.87)	5.30 (-0.18 -10.78)	0.054	
Normal weight	1.0 (Ref)	1.66 (-0.17 - 3.49)	8.12 (6.10 - 10.14)	0.000	
Overweight	1.0 (Ref)	-1.10 (-5.03 - 2.82)	2.81 (-1.31 - 6.92)	0.122	
Obese	1.0 (Ref)	0.13 (-5.47 - 5.73)	5.11 (-0.71 - 10.93)	0.080	
Presence of chronic disease in family					0.613
No	1.0 (Ref)	0.86 (-0.97 - 2.68)	6.86 (4.90 - 8.82)	0.000	
Yes	1.0 (Ref)	2.38 (-0.22 - 4.99)	8.40 (5.55 - 11.24)	0.000	
Caregiver education level					0.992
Junior high school or below	1.0 (Ref)	2.17 (-3.32 - 7.65)	7.56 (2.13 -12.99)	0.007	
Senior high school	1.0 (Ref)	1.70 (-1.18 - 4.58)	8.33 (5.32 - 11.35)	0.000	
College degree or above	1.0 (Ref)	1.41 (-0.48 - 3.30)	7.04 (4.97 - 9.11)	0.000	
Monthly household income					0.628

Variable	Group				
	Control group	Print media group	Short-video intervention group	*P* for Trend	*P* for Interaction
≤ 3,000 RMB	1.0 (Ref)	1.17 (-6.32 - 8.65)	9.74 (2.11 - 17.36)	0.007	
3,001-5,000 RMB	1.0 (Ref)	1.43 (-2.72 - 5.59)	6.50 (2.48 - 10.52)	0.002	
5,001–10,000 RMB	1.0 (Ref)	-0.70 (-3.84 - 2.45)	7.57 (4.18 - 10.95)	0.000	
> 10,000 RMB	1.0 (Ref)	2.23 (0.26 - 4.21)	7.09 (4.87 - 9.31)	0.000	
Caregiver occupation					0.013
Business / Service industry	1.0 (Ref)	-0.62 (-3.54 - 2.30)	7.47 (4.23 - 10.71)	0.000	
Public institution	1.0 (Ref)	4.71 (0.58 - 8.83)	6.03 (1.28 - 10.79)	0.015	
Industry / Manufacturing	1.0 (Ref)	-0.57 (-4.97 - 3.84)	9.20 (4.40 - 14.00)	0.000	
Other	1.0 (Ref)	1.96 (-0.24 - 4.16)	7.38 (5.10 - 9.66)	0.000	
Number of children cared for					0.673
1	1.0 (Ref)	2.19 (0.06 - 4.32)	7.43 (5.24 - 9.62)	0.000	
≥ 2	1.0 (Ref)	0.62 (-1.46 - 2.69)	7.00 (4.60 - 9.40)	0.000	
Relationship with the child					0.765
Mother-child	1.0 (Ref)	1.71 (0.16 - 3.26)	7.58 (5.88 - 9.28)	0.000	
Father-child	1.0 (Ref)	-1.63 (-7.86 - 4.60)	3.98 (-2.54 - 10.50)	0.078	
Other	1.0 (Ref)	-0.51 (-11.61 - 10.58)	1.23 (-9.03 - 11.49)	0.789	
Child sex					0.712
Male	1.0 (Ref)	0.86 (-1.13 - 2.85)	7.03 (4.82 - 9.24)	0.000	
Female	1.0 (Ref)	2.30 (0.06 - 4.53)	7.74 (5.35 - 10.13)	0.000	
Child age					0.5226
3-4 years	1.0 (Ref)	0.25 (-3.01 - 3.51)	6.07 (2.78 - 9.36)	0.001	
5-6 years	1.0 (Ref)	1.85 (0.18 - 3.53)	7.79 (5.95 - 9.64)	0.000	
Child BMI					0.667
Underweight	1.0 (Ref)	3.13 (-3.93 - 10.19)	9.02 (-0.12 - 18.16)	0.054	
Normal weight	1.0 (Ref)	2.40 (0.53 - 4.27)	7.47 (5.47 - 9.47)	0.000	
Overweight	1.0 (Ref)	-1.00 (-6.44 - 4.45)	8.66 (2.72 - 14.61)	0.005	
Obese	1.0 (Ref)	-1.11 (-4.38 - 2.16)	5.60 (2.03 - 9.18)	0.004	

Table S2. Subgroup analysis of knowledge scores among caregivers of preschool children at 1 week post-intervention

Variable	Group			*P* for Trend	*P* for Interaction
	Control group	Print media group	Short-video intervention group		
Caregiver sex					0.723
Male	1.0 (Ref)	1.22 (-6.17 - 8.62)	30.25 (21.99 - 38.52)	0.000	
Female	1.0 (Ref)	3.48 (-0.25 - 7.20)	28.70 (24.68 - 32.72)	0.000	
Caregiver age					0.061
≤ 30 years	1.0 (Ref)	8.57 (-0.30 -17.45)	29.98 (21.20 - 38.75)	0.000	
31-40 years	1.0 (Ref)	1.57 (-2.33 - 5.48)	29.92 (25.62 - 34.22)	0.000	
> 40 years	1.0 (Ref)	9.57 (0.08 - 19.07)	22.86 (12.85 - 32.87)	0.000	
Caregiver BMI					0.944
Underweight	1.0 (Ref)	-2.85 (-14.95 - 9.25)	19.28 (6.13 - 32.42)	0.007	
Normal weight	1.0 (Ref)	2.93 (-1.06 - 6.91)	29.37 (24.96 - 33.77)	0.000	
Overweight	1.0 (Ref)	2.87 (-6.48 - 12.21)	29.19 (19.40 - 38.98)	0.000	
Obese	1.0 (Ref)	3.05 (-9.95 - 16.06)	28.47 (14.95 - 41.99)	0.000	
Presence of chronic disease in family					0.523
No	1.0 (Ref)	2.06 (-1.98 - 6.10)	28.59 (24.25 - 32.92)	0.000	
Yes	1.0 (Ref)	5.10 (-0.97 - 11.18)	30.54 (23.92 - 37.17)	0.000	
Caregiver education level					0.910
Junior high school or below	1.0 (Ref)	7.55 (-4.36 - 19.47)	33.43 (21.64 - 45.23)	0.000	
Senior high school	1.0 (Ref)	2.99 (-4.23 - 10.21)	31.94 (24.40 - 39.49)	0.000	
College degree or above	1.0 (Ref)	2.84 (-1.24 - 6.93)	28.3 (23.90 - 32.85)	0.000	
Monthly household income					0.633
≤ 3,000 RMB	1.0 (Ref)	-8.44 (-24.63 - 7.76)	23.47 (6.97 - 39.97)	0.003	
3,001-5,000 RMB	1.0 (Ref)	4.20 (-6.77 - 15.17)	34.33 (23.70 - 44.96)	0.000	
5,001–10,000 RMB	1.0 (Ref)	0.86 (-5.83 - 7.55)	31.02 (23.82 - 38.22)	0.000	
> 10,000 RMB	1.0 (Ref)	4.32 (-0.00 - 8.65)	28.24 (23.38 - 33.10)	0.000	
Caregiver occupation					0.138
Business / Service industry	1.0 (Ref)	-0.24 (-6.65 - 6.18)	31.29 (24.17 -38.41)	0.000	
Public institution	1.0 (Ref)	7.27 (-0.98 - 15.52)	23.41 (13.88 - 32.94)	0.000	
Industry / Manufacturing	1.0 (Ref)	1.53 (-8.06 - 11.13)	32.25 (21.79 - 42.72)	0.000	
Other	1.0 (Ref)	2.77 (-2.41 - 7.94)	29.71 (24.34 - 35.08)	0.000	
Number of children cared for					0.200

Variable	Group				
	Control group	Print media group	Short-video intervention group	*P* for Trend	*P* for Interaction
1	1.0 (Ref)	1.97 (-2.80 - 6.75)	26.98 (22.08 - 31.88)	0.000	
≥ 2	1.0 (Ref)	5.11 (0.49 - 9.72)	33.13 (27.79 - 38.46)	0.000	
Relationship with the child					0.306
Mother-child	1.0 (Ref)	4.29 (0.77 - 7.82)	30.46 (26.59 - 34.33)	0.000	
Father-child	1.0 (Ref)	-6.57 (-19.19 - 6.04)	22.07 (8.87 - 35.27)	0.000	
Other	1.0 (Ref)	-6.15 (-29.57 - 17.28)	16.95 (-4.71 - 38.62)	0.101	
Child sex					0.317
Male	1.0 (Ref)	3.49 (-1.07 - 8.05)	27.56 (22.49 - 32.63)	0.000	
Female	1.0 (Ref)	2.64 (-2.20 - 7.47)	30.97 (25.81 - 36.12)	0.000	
Child age					0.328
3-4 years	1.0 (Ref)	-1.30 (-8.00 - 5.39)	29.58 (22.82 - 36.34)	0.000	
5-6 years	1.0 (Ref)	4.35 (0.48 - 8.22)	29.13 (24.87 - 33.38)	0.000	
Child BMI					0.862
Underweight	1.0 (Ref)	-2.37 (-18.56 - 13.83)	39.77 (18.81 - 60.74)	0.007	
Normal weight	1.0 (Ref)	4.02 (-0.19 - 8.23)	29.17 (24.67 - 33.68)	0.000	
Overweight	1.0 (Ref)	3.66 (-8.03 - 15.34)	34.09 (21.34 - 46.85)	0.000	
Obese	1.0 (Ref)	3.12 (-3.89 - 10.13)	27.39 (19.73 - 35.05)	0.000	

Table S3. Subgroup analysis of attitude scores among caregivers of preschool children at 1 week post-intervention

Variable	Group				
	**Control group**	**Print media group**	**Short-video intervention group**	***P* for Trend**	***P* for Interaction**
Caregiver sex					0.126
Male	1.0 (Ref)	-2.76 (-8.62 - 3.10)	6.92 (0.37 - 13.46)	0.027	
Female	1.0 (Ref)	2.48 (-0.84 - 5.80)	6.70 (3.12 - 10.28)	0.000	
Caregiver age					0.310
≤ 30 years	1.0 (Ref)	3.52 (-4.30 - 11.34)	2.74 (-4.99 - 10.48)	0.488	
31-40 years	1.0 (Ref)	0.39 (-3.04 - 3.81)	7.89 (4.12 - 11.65)	0.000	
> 40 years	1.0 (Ref)	6.64 (-1.93 - 15.21)	6.25 (-2.79 - 15.29)	0.179	
Caregiver BMI					0.988
Underweight	1.0 (Ref)	5.42 (-4.94 - 15.78)	4.06 (-7.20 - 15.32)	0.456	
Normal weight	1.0 (Ref)	2.27 (-1.31 - 5.86)	7.28 (3.33 - 11.24)	0.000	
Overweight	1.0 (Ref)	-0.57 (-8.61 - 7.47)	4.68 (-3.75 - 13.11)	0.224	
Obese	1.0 (Ref)	-3.71 (-15.03 - 7.62)	2.71 (-9.06 - 14.48)	0.630	
Presence of chronic disease in family					0.400
No	1.0 (Ref)	0.42 (-3.21 - 4.04)	5.26 (1.36 - 9.15)	0.007	
Yes	1.0 (Ref)	3.70 (-1.22 - 8.63)	10.68 (5.31 - 16.05)	0.000	
Caregiver education level					0.984
Junior high school or below	1.0 (Ref)	0.84 (-6.71 - 8.38)	6.86 (-0.61 - 14.32)	0.074	
Senior high school	1.0 (Ref)	1.67 (-4.35 - 7.69)	8.23 (1.94 - 14.52)	0.010	
College degree or above	1.0 (Ref)	1.77 (-1.95 - 5.50)	6.75 (2.67 - 10.83)	0.001	
Monthly household income					0.781
≤ 3,000 RMB	1.0 (Ref)	7.97 (-7.11 - 23.05)	11.23 (-4.15 - 26.60)	0.157	
3,001-5,000 RMB	1.0 (Ref)	3.11 (-5.65 - 11.87)	5.42 (-3.08 - 13.91)	0.207	
5,001–10,000 RMB	1.0 (Ref)	-0.97 (-6.88 - 4.94)	8.08 (1.71 - 14.44)	0.013	
> 10,000 RMB	1.0 (Ref)	1.58 (-2.32 - 5.47)	6.24 (1.86 - 10.63)	0.005	
Caregiver occupation					0.247
Business / Service industry	1.0 (Ref)	-0.33 (-5.79 - 5.13)	4.30 (-1.76 - 10.36)	0.174	
Public institution	1.0 (Ref)	4.32 (-4.08 - 12.73)	3.38 (-6.32 - 13.09)	0.514	
Industry / Manufacturing	1.0 (Ref)	1.15 (-7.09 - 9.39)	11.54 (2.56 - 20.53)	0.010	
Other	1.0 (Ref)	1.81 (-2.61 - 6.23)	7.67 (3.08 - 12.27)	0.001	
Number of children cared for					0.186

Variable	Group				
	Control group	Print media group	Short-video intervention group	*P* for Trend	*P* for Interaction
1	1.0 (Ref)	3.89 (-0.42 - 8.21)	8.00 (3.57 - 12.43)	0.000	
≥ 2	1.0 (Ref)	-1.36 (-5.19 - 2.48)	5.03 (0.60 - 9.46)	0.030	
Relationship with the child					0.963
Mother-child	1.0 (Ref)	0.99 (-2.12 - 4.11)	6.51 (3.09 - 9.93)	0.000	
Father-child	1.0 (Ref)	2.41 (-8.75 - 13.58)	7.17 (-4.52 - 18.85)	0.170	
Other	1.0 (Ref)	0.97 (-17.52 - 19.46)	1.82 (-15.29 - 18.92)	0.826	
Child sex					0.071
Male	1.0 (Ref)	-1.80 (-5.89 - 2.30)	4.54 (-0.01 - 9.10)	0.050	
Female	1.0 (Ref)	5.71 (1.61 - 9.82)	10.24 (5.86 - 14.62)	0.000	
Child age					0.598
3-4 years	1.0 (Ref)	3.11 (-3.34 - 9.55)	6.30 (-0.21 - 12.81)	0.057	
5-6 years	1.0 (Ref)	0.84 (-2.43 - 4.11)	7.29 (3.69 - 10.90)	0.000	
Child BMI					0.806
Underweight	1.0 (Ref)	0.83 (-17.73 - 19.40)	-1.62 (-25.65 - 22.42)	0.924	
Normal weight	1.0 (Ref)	2.47 (-1.15 - 6.09)	6.97 (3.09 - 10.85)	0.000	
Overweight	1.0 (Ref)	-2.79 (-13.55 - 7.96)	5.15 (-6.58 - 16.89)	0.378	
Obese	1.0 (Ref)	-0.10 (-6.29 - 6.10)	7.36 (0.59 - 14.12)	0.038	

Table S4. Subgroup analysis of practice scores among caregivers of preschool children at 1 week post-intervention

Variable	Group				
	Control group	Print media group	Short-video intervention group	*P* for Trend	*P* for Interaction
Caregiver sex					0.212
Male	1.0 (Ref)	0.72 (-2.83 - 4.27)	5.50 (1.54 - 9.47)	0.005	
Female	1.0 (Ref)	1.48 (-0.28 - 3.24)	3.36 (1.46 - 5.26)	0.001	
Caregiver age					0.257
≤ 30 years	1.0 (Ref)	2.44 (-2.16 - 7.04)	3.46 (-1.08 - 8.01)	0.134	
31-40 years	1.0 (Ref)	0.06 (-1.72 - 1.83)	3.37 (1.41 - 5.32)	0.001	
> 40 years	1.0 (Ref)	3.95 (-1.11 - 9.02)	3.46 (-1.87 - 8.80)	0.208	
Caregiver BMI					0.261
Underweight	1.0 (Ref)	4.29 (-1.00 - 9.58)	3.47 (-2.28 - 9.21)	0.218	
Normal weight	1.0 (Ref)	1.20 (-0.78 - 3.18)	4.92 (2.73 - 7.10)	0.000	
Overweight	1.0 (Ref)	-1.98 (-5.80 - 1.84)	-2.34 (-6.34 - 1.66)	0.274	
Obese	1.0 (Ref)	1.18 (-4.85 - 7.21)	2.18 (-4.10 - 8.45)	0.489	
Presence of chronic disease in family					0.760
No	1.0 (Ref)	0.83 (-1.10 - 2.76)	3.88 (1.81 - 5.95)	0.000	
Yes	1.0 (Ref)	1.40 (-1.40 - 4.21)	3.79 (0.74 - 6.85)	0.014	
Caregiver education level					0.999
Junior high school or below	1.0 (Ref)	1.80 (-4.24 - 7.84)	3.53 (-2.45 - 9.51)	0.240	
Senior high school	1.0 (Ref)	1.50 (-1.83 - 4.83)	4.44 (0.97 - 7.92)	0.012	
College degree or above	1.0 (Ref)	1.03 (-0.94 - 2.99)	3.60 (1.45 - 5.75)	0.001	
Monthly household income					0.654
≤ 3,000 RMB	1.0 (Ref)	0.05 (-7.84 - 7.93)	6.86 (-1.18 - 14.89)	0.060	
3,001-5,000 RMB	1.0 (Ref)	0.30 (-4.50 - 5.10)	2.30 (-2.36 - 6.95)	0.326	
5,001–10,000 RMB	1.0 (Ref)	-0.85 (-4.11 - 2.41)	3.45 (-0.06 - 6.96)	0.054	
> 10,000 RMB	1.0 (Ref)	2.15 (0.05 - 4.25)	3.91 (1.55 - 6.27)	0.001	
Caregiver occupation					0.068
Business / Service industry	1.0 (Ref)	-0.80 (-4.15 - 2.55)	4.77 (1.05 - 8.48)	0.016	
Public institution	1.0 (Ref)	4.43 (0.14 - 8.72)	4.19 (-0.76 - 9.15)	0.110	
Industry / Manufacturing	1.0 (Ref)	-1.60 (-5.92 - 2.71)	4.42 (-0.29 - 9.13)	0.053	
Other	1.0 (Ref)	1.88 (-0.43 - 4.20)	3.54 (1.13 - 5.94)	0.004	
Number of children cared for					0.933

Variable	Group				
	Control group	Print media group	Short-video intervention group	*P* for Trend	*P* for Interaction
1	1.0 (Ref)	1.54 (-0.68 - 3.77)	3.94 (1.66 - 6.22)	0.001	
≥ 2	1.0 (Ref)	0.66 (-1.60 - 2.92)	3.43 (0.82 - 6.04)	0.011	
Relationship with the child					0.777
Mother-child	1.0 (Ref)	1.57 (-0.10 - 3.23)	4.19 (2.36 - 6.02)	0.000	
Father-child	1.0 (Ref)	-2.42 (-8.54 - 3.70)	-0.31 (-6.71 - 6.09)	0.817	
Other	1.0 (Ref)	-0.17 (-10.48 - 10.14)	-1.63 (-11.17 - 7.91)	0.714	
Child sex					0.673
Male	1.0 (Ref)	1.48 (-0.65 - 3.62)	4.61 (2.23 - 6.98)	0.000	
Female	1.0 (Ref)	0.87 (-1.49 - 3.23)	2.87 (0.35 - 5.39)	0.024	
Child age					0.271
3-4 years	1.0 (Ref)	-0.63 (-3.93 - 2.67)	2.06 (-1.28 - 5.39)	0.260	
5-6 years	1.0 (Ref)	1.84 (0.03 - 3.65)	4.44 (2.44 - 6.44)	0.000	
Child BMI					0.483
Underweight	1.0 (Ref)	4.97 (-1.98 - 11.92)	8.16 (-0.84 - 17.15)	0.053	
Normal weight	1.0 (Ref)	2.11 (0.12 - 4.09)	4.05 (1.92 - 6.18)	0.000	
Overweight	1.0 (Ref)	-1.05 (-6.80 - 4.70)	5.83 (-0.45 - 12.10)	0.068	
Obese	1.0 (Ref)	-2.22 (-5.77 - 1.33)	1.27 (-2.60 - 5.15)	0.565	

Table S5. Subgroup analysis of total KAP scores among caregivers of preschool children at 2 months post-intervention

Variable	Group			*P* for Trend	*P* for Interaction
	Control group	Print media group	Short-video intervention group		
Caregiver sex					0.255
Male	1.0 (Ref)	-2.21 (-5.85 - 1.42)	2.94 (-1.12 - 7.00)	0.116	
Female	1.0 (Ref)	1.41 (-0.36 - 3.19)	5.71 (3.80 - 7.62)	0.000	
Caregiver age					0.194
≤ 30 years	1.0 (Ref)	-1.26 (-5.21 - 2.68)	2.99 (-0.91 - 6.90)	0.129	
31-40 years	1.0 (Ref)	0.11 (-1.75 - 1.97)	5.31 (3.26 - 7.36)	0.000	
> 40 years	1.0 (Ref)	5.99 (0.58 - 11.39)	7.27 (1.57 - 12.97)	0.014	
Caregiver BMI					0.421
Underweight	1.0 (Ref)	3.33 (-2.49 - 9.16)	2.41 (-3.92 - 8.73)	0.432	
Normal weight	1.0 (Ref)	0.55 (-1.44 - 2.54)	5.87 (3.68 - 8.06)	0.000	
Overweight	1.0 (Ref)	1.25 (-2.84 - 5.34)	3.38 (-0.90 - 7.67)	0.109	
Obese	1.0 (Ref)	-3.62 (-9.43 - 2.19)	3.02 (-3.01 - 9.06)	0.308	
Presence of chronic disease in family					0.586
No	1.0 (Ref)	0.77 (-1.16 - 2.69)	5.74 (3.67 - 7.81)	0.000	
Yes	1.0 (Ref)	0.56 (-2.33 - 3.45)	4.45 (1.29 - 7.60)	0.005	
Caregiver education level					0.876
Junior high school or below	1.0 (Ref)	-0.82 (-6.46 - 4.82)	3.06 (-2.52 - 8.64)	0.290	
Senior high school	1.0 (Ref)	0.55 (-2.75 - 3.85)	5.50 (2.05 - 8.95)	0.002	
College degree or above	1.0 (Ref)	1.06 (-0.94 - 3.06)	5.83 (3.64 - 8.02)	0.000	
Monthly household income					0.541
≤ 3,000 RMB	1.0 (Ref)	3.14 (-4.33 - 10.61)	3.51 (-4.10 - 11.12)	0.389	
3,001-5,000 RMB	1.0 (Ref)	-1.64 (-6.40 - 3.12)	4.52 (-0.09 - 9.14)	0.055	
5,001–10,000 RMB	1.0 (Ref)	-0.77 (-3.88 - 2.35)	6.32 (2.96 - 9.67)	0.000	
> 10,000 RMB	1.0 (Ref)	1.08 (-1.05 - 3.22)	5.44 (3.04 - 7.85)	0.000	
Caregiver occupation					0.161
Business / Service industry	1.0 (Ref)	1.62 (-1.47 - 4.71)	8.73 (5.30 - 12.16)	0.000	
Public institution	1.0 (Ref)	0.99 (-3.06 - 5.05)	3.71 (-0.97 - 8.39)	0.112	
Industry / Manufacturing	1.0 (Ref)	-0.71 (-5.40 - 3.97)	7.42 (2.31 - 12.53)	0.004	
Other	1.0 (Ref)	1.01 (-1.42 - 3.45)	4.09 (1.56 - 6.62)	0.001	
Number of children cared for					0.451

Variable	Group				
	Control group	Print media group	Short-video intervention group	*P* for Trend	*P* for Interaction
1	1.0 (Ref)	0.74 (-1.43 - 2.92)	6.11 (3.88 - 8.35)	0.000	
≥ 2	1.0 (Ref)	0.62 (-1.72 - 2.97)	4.14 (1.42 - 6.85)	0.003	
Relationship with the child					0.258
Mother-child	1.0 (Ref)	0.55 (-1.13 - 2.24)	5.52 (3.67 - 7.37)	0.000	
Father-child	1.0 (Ref)	2.74 (-3.24 - 8.73)	6.13 (-0.13 - 12.39)	0.038	
Other	1.0 (Ref)	-1.37 (-12.52 - 9.78)	-4.67 (-14.98 - 5.64)	0.343	
Child sex					0.584
Male	1.0 (Ref)	0.52 (-1.71 - 2.74)	4.76 (2.28 - 7.23)	0.000	
Female	1.0 (Ref)	1.41 (-0.88 - 3.70)	6.53 (4.08 - 8.97)	0.000	
Child age					0.109
3-4 years	1.0 (Ref)	-3.03 (-6.23 - 0.18)	4.25 (1.02 - 7.49)	0.025	
5-6 years	1.0 (Ref)	1.78 (-0.05 - 3.62)	5.90 (3.88 - 7.92)	0.000	
Child BMI					0.232
Underweight	1.0 (Ref)	1.86 (-6.81 - 10.54)	2.79 (-8.45 - 14.02)	0.578	
Normal weight	1.0 (Ref)	0.41 (-1.53 - 2.36)	5.05 (2.97 - 7.13)	0.000	
Overweight	1.0 (Ref)	5.60 (0.03 - 11.17)	9.84 (3.76 - 15.92)	0.002	
Obese	1.0 (Ref)	-0.87 (-4.47 - 2.72)	3.26 (-0.67 - 7.19)	0.118	

Table S6. Subgroup analysis of knowledge scores among caregivers of preschool children at 2 months post-intervention

Variable	Group			*P* for Trend	*P* for Interaction
	Control group	Print media group	Short-video intervention group		
Caregiver sex					0.817
Male	1.0 (Ref)	1.74 (-6.36 - 9.85)	16.35 (7.30 - 25.40)	0.000	
Female	1.0 (Ref)	0.80 (-3.13 - 4.73)	16.73 (12.49 - 20.97)	0.000	
Caregiver age					0.233
≤ 30 years	1.0 (Ref)	4.03 (-5.71 - 13.77)	17.99 (8.36 - 27.61)	0.000	
31-40 years	1.0 (Ref)	-1.27 (-5.42 - 2.87)	15.88 (11.32 - 20.44)	0.000	
> 40 years	1.0 (Ref)	14.14 (3.39 -24.89)	22.64 (11.31 - 33.96)	0.000	
Caregiver BMI					0.956
Underweight	1.0 (Ref)	3.85 (-9.54 - 17.23)	9.45 (-5.09 - 23.99)	0.198	
Normal weight	1.0 (Ref)	1.70 (-2.67 - 6.07)	18.83 (14.01 - 23.66)	0.000	
Overweight	1.0 (Ref)	-4.23 (-13.08 - 4.63)	11.51 (2.23 - 20.79)	0.006	
Obese	1.0 (Ref)	-2.31 (-14.80 - 10.18)	14.19 (1.21 - 27.18)	0.032	
Presence of chronic disease in family					0.501
No	1.0 (Ref)	0.02 (-4.28 - 4.32)	17.33 (12.72 - 21.95)	0.000	
Yes	1.0 (Ref)	2.86 (-3.54 - 9.26)	16.04 (9.06 - 23.03)	0.000	
Caregiver education level					0.251
Junior high school or below	1.0 (Ref)	4.34 (-7.14 - 15.82)	23.06 (11.70 - 34.43)	0.000	
Senior high school	1.0 (Ref)	-2.84 (-10.54 - 4.87)	11.31 (3.26 - 19.35)	0.006	
College degree or above	1.0 (Ref)	3.15 (-1.14 - 7.44)	19.81 (15.11 - 24.51)	0.000	
Monthly household income					0.139
≤ 3,000 RMB	1.0 (Ref)	-2.57 (-22.23 - 17.09)	6.08 (-13.95 - 26.11)	0.456	
3,001-5,000 RMB	1.0 (Ref)	-3.68 (-14.39 - 7.03)	18.23 (7.85 - 28.62)	0.001	
5,001–10,000 RMB	1.0 (Ref)	-2.93 (-9.82 - 3.97)	16.61 (9.18 - 24.03)	0.000	
> 10,000 RMB	1.0 (Ref)	3.75 (-0.82 - 8.31)	18.99 (13.86 - 24.12)	0.000	
Caregiver occupation					0.211
Business / Service industry	1.0 (Ref)	2.93 (-3.44 - 9.30)	24.67 (17.60 - 31.74)	0.000	
Public institution	1.0 (Ref)	6.36 (-1.69 - 14.41)	19.08 (9.79 - 28.37)	0.000	
Industry / Manufacturing	1.0 (Ref)	-0.58 (-10.98 - 9.81)	20.77 (9.44 - 32.11)	0.000	
Other	1.0 (Ref)	0.27 (-5.48 - 6.02)	13.06 (7.08 - 19.03)	0.000	
Number of children cared for					0.403

Variable	Group				
	Control group	Print media group	Short-video intervention group	*P* for Trend	*P* for Interaction
1	1.0 (Ref)	0.69 (-4.02 - 5.41)	18.96 (14.12 - 23.81)	0.000	
≥ 2	1.0 (Ref)	1.53 (-3.81 - 6.87)	15.22 (9.04 - 21.39)	0.000	
Relationship with the child					0.433
Mother-child	1.0 (Ref)	0.66 (-3.13 - 4.45)	17.10 (12.94 - 21.26)	0.000	
Father-child	1.0 (Ref)	9.00 (-3.20 - 21.19)	20.83 (8.07 - 33.59)	0.001	
Other	1.0 (Ref)	-1.76 (-25.01 - 21.49)	15.95 (-5.56 - 37.46)	0.119	
Child sex					0.251
Male	1.0 (Ref)	1.77 (-3.12 - 6.67)	15.65 (10.20 - 21.09)	0.000	
Female	1.0 (Ref)	1.13 (-4.03 - 6.29)	20.23 (14.72 - 25.73)	0.000	
Child age					0.473
3-4 years	1.0 (Ref)	-2.17 (-9.56 - 5.21)	15.33 (7.88 - 22.79)	0.000	
5-6 years	1.0 (Ref)	2.51 (-1.57 - 6.59)	17.91 (13.42 - 22.40)	0.000	
Child BMI					0.934
Underweight	1.0 (Ref)	4.19 (-14.04 - 22.41)	15.46 (-8.13 - 39.05)	0.205	
Normal weight	1.0 (Ref)	1.77 (-2.65 - 6.19)	17.18 (12.45 - 21.91)	0.000	
Overweight	1.0 (Ref)	5.56 (-7.12 - 18.24)	23.16 (9.32 - 37.00)	0.001	
Obese	1.0 (Ref)	0.54 (-7.22 - 8.31)	16.50 (8.01 - 24.99)	0.000	

Table S7. Subgroup analysis of attitude scores among caregivers of preschool children at 2 months post-intervention

Variable	Group				
	Control group	Print media group	Short-video intervention group	*P* for Trend	*P* for Interaction
Caregiver sex					0.730
Male	1.0 (Ref)	-1.70 (-8.32 - 4.92)	3.75 (-3.65 - 11.14)	0.277	
Female	1.0 (Ref)	2.50 (-0.90 - 5.91)	7.71 (4.03 - 11.38)	0.000	
Caregiver age					0.664
≤ 30 years	1.0 (Ref)	-1.68 (-9.20 - 5.84)	6.31 (-1.12 - 13.74)	0.093	
31-40 years	1.0 (Ref)	1.47 (-2.11 - 5.06)	7.12 (3.18 - 11.07)	0.000	
> 40 years	1.0 (Ref)	8.26 (-1.23 - 17.76)	7.65 (-2.36 - 17.66)	0.139	
Caregiver BMI					0.554
Underweight	1.0 (Ref)	3.19 (-9.11 - 15.48)	3.14 (-10.22 - 16.49)	0.631	
Normal weight	1.0 (Ref)	2.96 (-0.78 - 6.69)	8.04 (3.92 - 12.16)	0.000	
Overweight	1.0 (Ref)	3.08 (-4.29 - 10.45)	4.71 (-3.02 - 12.44)	0.237	
Obese	1.0 (Ref)	-9.57 (-21.03 - 1.90)	1.66 (-10.26 - 13.58)	0.749	
Presence of chronic disease in family					0.539
No	1.0 (Ref)	1.02 (-2.67 - 4.72)	7.52 (3.55 - 11.49)	0.000	
Yes	1.0 (Ref)	2.49 (-2.87 - 7.84)	5.86 (0.01 - 11.70)	0.048	
Caregiver education level					0.980
Junior high school or below	1.0 (Ref)	1.76 (-8.11 - 11.63)	3.83 (-5.94 - 13.60)	0.434	
Senior high school	1.0 (Ref)	2.37 (-4.10 - 8.83)	9.23 (2.47 - 15.98)	0.007	
College degree or above	1.0 (Ref)	1.72 (-2.05 - 5.49)	7.24 (3.11 - 11.37)	0.000	
Monthly household income					0.228
≤ 3,000 RMB	1.0 (Ref)	8.12 (-7.55 - 23.79)	4.33 (-11.64 - 20.30)	0.701	
3,001-5,000 RMB	1.0 (Ref)	-2.43 (-11.69 - 6.83)	3.83 (-5.14 - 12.80)	0.393	
5,001–10,000 RMB	1.0 (Ref)	2.06 (-4.25 - 8.36)	11.24 (4.46 - 18.03)	0.001	
> 10,000 RMB	1.0 (Ref)	0.47 (-3.42 - 4.37)	6.30 (1.92 - 10.68)	0.005	
Caregiver occupation					0.555
Business / Service industry	1.0 (Ref)	1.06 (-5.13 - 7.26)	9.81 (2.93 - 16.69)	0.006	
Public institution	1.0 (Ref)	0.22 (-7.50 - 7.95)	2.87 (-6.05 - 11.78)	0.512	
Industry / Manufacturing	1.0 (Ref)	-0.37 (-8.37 - 7.63)	9.60 (0.88 - 18.32)	0.026	
Other	1.0 (Ref)	3.86 (-0.83 - 8.55)	7.08 (2.21 - 11.95)	0.004	
Number of children cared for					0.790

Variable	Group				
	Control group	Print media group	Short-video intervention group	*P* for Trend	*P* for Interaction
1	1.0 (Ref)	1.01 (-3.03 - 5.05)	7.12 (2.97 - 11.27)	0.001	
≥ 2	1.0 (Ref)	2.91 (-1.62 - 7.44)	6.78 (1.54 - 12.01)	0.011	
Relationship with the child					0.188
Mother-child	1.0 (Ref)	1.04 (-2.22 - 4.30)	7.28 (3.71 - 10.86)	0.000	
Father-child	1.0 (Ref)	13.23 (2.31 - 24.14)	12.40 (0.98 - 23.82)	0.100	
Other	1.0 (Ref)	0.92 (-14.94 - 16.78)	-4.17 (-18.84 - 10.51)	0.534	
Child sex					0.247
Male	1.0 (Ref)	3.95 (-0.17 - 8.07)	7.81 (3.23 - 12.39)	0.001	
Female	1.0 (Ref)	-0.14 (-4.64 - 4.36)	6.85 (2.04 - 11.65)	0.004	
Child age					0.624
3-4 years	1.0 (Ref)	-1.84 (-7.63 - 3.95)	6.96 (1.11 - 12.80)	0.031	
5-6 years	1.0 (Ref)	2.55 (-1.00 - 6.10)	7.17 (3.27 - 11.08)	0.000	
Child BMI					0.582
Underweight	1.0 (Ref)	4.32 (-13.74 - 22.38)	3.76 (-19.61 - 27.14)	0.678	
Normal weight	1.0 (Ref)	1.38 (-2.44 - 5.19)	6.14 (2.05 - 10.22)	0.003	
Overweight	1.0 (Ref)	7.26 (-3.25 - 17.77)	11.47 (0.00 - 22.94)	0.049	
Obese	1.0 (Ref)	-1.14 (-7.27 - 4.98)	5.67 (-1.02 - 12.37)	0.108	

Table S8. Subgroup analysis of practice scores among caregivers of preschool children at 2 months post-intervention

Variable	Group				
	Control group	Print media group	Short-video intervention group	*P* for Trend	*P* for Interaction
Caregiver sex					0.082
Male	1.0 (Ref)	-3.08 (-6.95 - 0.79)	0.38 (-3.93 - 4.70)	0.754	
Female	1.0 (Ref)	1.08 (-0.69 - 2.85)	3.08 (1.17 - 4.98)	0.002	
Caregiver age					0.234
≤ 30 years	1.0 (Ref)	-1.98 (-6.22 - 2.26)	-0.83 (-5.02 - 3.36)	0.703	
31-40 years	1.0 (Ref)	-0.21 (-2.05 - 1.64)	2.82 (0.79 - 4.86)	0.005	
> 40 years	1.0 (Ref)	3.72 (-1.72 - 9.16)	4.55 (-1.18 - 10.29)	0.120	
Caregiver BMI					0.440
Underweight	1.0 (Ref)	3.31 (-1.79 - 8.40)	0.94 (-4.60 - 6.48)	0.702	
Normal weight	1.0 (Ref)	-0.61 (-2.65 - 1.43)	2.85 (0.59 - 5.10)	0.012	
Overweight	1.0 (Ref)	1.44 (-2.80 - 5.68)	1.50 (-2.95 - 5.94)	0.536	
Obese	1.0 (Ref)	-1.46 (-7.46 - 4.54)	1.70 (-4.53 - 7.94)	0.572	
Presence of chronic disease in family					0.701
No	1.0 (Ref)	0.79 (-1.18 - 2.75)	3.09 (0.98 - 5.21)	0.004	
Yes	1.0 (Ref)	-0.59 (-3.42 - 2.24)	1.95 (-1.14 - 5.04)	0.199	
Caregiver education level					0.772
Junior high school or below	1.0 (Ref)	-2.71 (-8.56 - 3.14)	-0.58 (-6.37 - 5.21)	0.820	
Senior high school	1.0 (Ref)	0.39 (-3.04 - 3.81)	3.04 (-0.54 - 6.61)	0.094	
College degree or above	1.0 (Ref)	0.44 (-1.56 - 2.44)	2.94 (0.75 - 5.13)	0.007	
Monthly household income					0.973
≤ 3,000 RMB	1.0 (Ref)	2.10 (-6.13 - 10.34)	2.75 (-5.64 - 11.15)	0.526	
3,001-5,000 RMB	1.0 (Ref)	-0.99 (-5.77 - 3.80)	2.51 (-2.12 - 7.15)	0.280	
5,001–10,000 RMB	1.0 (Ref)	-1.54 (-4.70 - 1.63)	2.63 (-0.77 - 6.04)	0.131	
> 10,000 RMB	1.0 (Ref)	0.89 (-1.29 - 3.07)	2.84 (0.39 - 5.29)	0.022	
Caregiver occupation					0.443
Business / Service industry	1.0 (Ref)	1.63 (-1.50 - 4.76)	5.64 (2.17 - 9.12)	0.002	
Public institution	1.0 (Ref)	0.41 (-3.88 - 4.69)	1.49 (-3.46 - 6.43)	0.545	
Industry / Manufacturing	1.0 (Ref)	-0.87 (-5.39 - 3.64)	4.32 (-0.60 - 9.25)	0.071	
Other	1.0 (Ref)	-0.01 (-2.47 - 2.46)	1.40 (-1.16 - 3.95)	0.268	
Number of children cared for					0.438

Variable	Group<br>Control group	Print media group	Short-video intervention group	*P* for Trend	*P* for Interaction
1	1.0 (Ref)	0.64 (-1.63 - 2.92)	3.56 (1.23 - 5.90)	0.002	
≥ 2	1.0 (Ref)	-0.44 (-2.72 - 1.84)	1.23 (-1.41 - 3.87)	0.372	
Relationship with the child					0.441
Mother-child	1.0 (Ref)	0.34 (-1.34 - 2.02)	2.89 (1.04 - 4.74)	0.002	
Father-child	1.0 (Ref)	-2.49 (-9.12 - 4.13)	1.18 (-5.76 - 8.11)	0.442	
Other	1.0 (Ref)	-2.22 (-14.75 - 10.30)	-8.31 (-19.90 - 3.28)	0.138	
Child sex					0.223
Male	1.0 (Ref)	-1.07 (-3.32 - 1.19)	1.72 (-0.78 - 4.23)	0.174	
Female	1.0 (Ref)	2.07 (-0.22 - 4.37)	4.11 (1.66 - 6.57)	0.001	
Child age					0.084
3-4 years	1.0 (Ref)	-3.65 (-7.00 --0.29)	1.33 (-2.06 - 4.71)	0.592	
5-6 years	1.0 (Ref)	1.36 (-0.48 - 3.19)	3.39 (1.37 - 5.41)	0.001	
Child BMI					0.279
Underweight	1.0 (Ref)	0.49 (-9.02 - 10.01)	0.29 (-12.03 - 12.61)	0.945	
Normal weight	1.0 (Ref)	-0.20 (-2.13 - 1.73)	2.60 (0.53 - 4.67)	0.010	
Overweight	1.0 (Ref)	4.94 (-1.20 - 11.07)	6.98 (0.28 - 13.67)	0.041	
Obese	1.0 (Ref)	-1.00 (-4.70 - 2.70)	0.09 (-3.96 - 4.13)	0.985	
