# Supplementary material for: Effects of Salt-Reduction Health Education Short Videos on Salt-Related Knowledge, Attitudes, and Practices (KAP) Among Caregivers of Preschool Children
Source: Nutrients. 2026 Oct 26;18(21):3525. doi: 10.3390/nu18213525 (PMC13764901; doi:10.3390/nu18213525)
Supplement: Supplementary file 1 [file nutrients-18-03525-s001.zip › Suppl. Mat. File S1 - The questionnaire for this study..pdf]

Questionnaire Code: Survey Date: Survey Location: Surveyor:

Survey on Salt Intake status and Salt-Reduction Knowledge, Attitudes, and Practices among Caregivers of Preschool Children

Informed Consent Form

Dear Parents/Caregivers of Preschool Children:

With changes in lifestyle, excessive salt intake has become an important public health issue affecting residents' health. Long-term excessive salt intake not only increases the risk of hypertension but may also increase the risk of various chronic diseases, such as cardiovascular diseases and stroke. Childhood is a critical period for the formation of dietary habits, and the family dietary environment and caregivers' dietary behaviors play an important role in shaping children's dietary habits. Therefore, improving caregivers' salt-reduction-related knowledge and promoting positive attitudes and behaviors toward salt reduction are of great significance for children's healthy growth.

This study is conducted by Chongqing Medical University. The purpose of this study is to investigate the current status of salt-related knowledge, attitudes, and practices among caregivers of preschool children and to explore the effects of different health education approaches (salt-reduction health education short videos and traditional print-based health education materials) on caregivers' salt-related knowledge, attitudes, and practices, thereby providing evidence for the development of more scientific and effective family-based salt reduction health education programs for children in the future.

If you agree to participate in this study, you will be asked to complete a questionnaire according to the requirements of the study. According to the study design, some participants will receive salt-reduction health education materials provided by the research team. The researchers will evaluate changes in caregivers' salt-related knowledge, attitudes, and practices before and after the health education intervention based on the questionnaire results.

This study has been reviewed and approved by the Ethics Committee of Chongqing Medical University. Your participation is entirely voluntary, and you may decide whether to participate in this study according to your own willingness. All information collected during this study will be used solely for scientific research purposes, and the research team will strictly protect your personal information and survey data.

This questionnaire is expected to take approximately 5–10 minutes to complete. There are no right or wrong answers. Please complete each item based on your actual situation, select the option that best reflects your true circumstances, and do not omit any questions. For questions with multiple options, please select one or more answers according to the instructions and your actual situation.

Thank you for your cooperation and support!

I have read and understood the information provided above. After careful consideration, I voluntarily agree to participate in this survey.

☐ Yes ☐ No (Skip to the end of the questionnaire and submit)

I. Basic Information

(Ⅰ) Basic Information of Caregivers

1. sex: A Male B Female
2. Age: ______ years
3.Your height is: ______ Centimeters, for example, 170 cm
4.Your weight is: ______ Kilograms, for example, 60 kg
5. Does anyone in your household (including yourself) have the following chronic diseases?
A Hypertension B Diabetes mellitus C Hyperlipidemia D Coronary heart disease E Stroke
F Other: ______ G None
6. your education level:
A Primary school or below B Junior high school C Senior high school / Vocational high school / Technical secondary school D Undergraduate degree / College degree E Master's degree or above
7. How many permanent residents are there in your household? ______ persons (including domestic helpers employed for more than 6 months)
8. What is the average total monthly income of your family (including income from sources such as housing leasing)?
A ≤ 3,000 RMB B 3,001-5,000 RMB C 5,001-10,000 RMB D ≥ 10,000 RMB
9. What is your occupation (if you are retired or unemployed, please list your most significant former employer)？
A Business / Service industry B Public institution C Industry / Manufacturing D Other: ______
10. How many children (aged 0-18 years) are you currently the primary caregiver for?
A 1 child B 2 children C 3 or more children
11. What is your relationship with the child you are caring for?
A Mother B Father C Grandparent D Other: ______

(Ⅱ) Basic information about your child (Please provide information for only one child aged 3-6 years)

12. Which class is your child currently enrolled in? ______
13. Child's name: ______
14. Child's sex: A Male B Female
15. Child's date of birth (Gregorian calendar): ____ Year ____ Month ____ Day
16. Height of your child: ______ Centimeters, for example, 170 cm
17. Weight of your child: ______ Kilograms, for example, 60 kg

II. Knowledge of Salt and Health

1. How would you describe the taste preference of your usual diet?
A Salty B Moderate C Light
2. Do you think that eating less salt can cause people to feel weak?
A Yes B No C Don't know
3. What is the maximum recommended daily salt intake for preschool children?
A 2 g B 3 g C 4 g D 5 g E 6 g F Don't know
4. What is the maximum recommended daily salt intake for adults?
A 2 g B 3 g C 4 g D 5 g E 6 g F Don't know
5. Excessive salt intake over a long period may increase the risk of which diseases? (Multiple choices allowed)
A Hypertension B Gastric cancer C Obesity D Osteoporosis E Stroke F Kidney disease
G Don't know
6. Which of the following snacks do you think do not contain salt? (Multiple choices allowed)
A Potato chips B Ham sausage C Soda crackers D Preserved plums E Cheese sticks
F Instant crispy noodles G None H Don't know
7. Which of the following foods and seasonings do you think do not contain salt? (Multiple choices allowed)

A Pickled foods B Dried noodles C Monosodium glutamate (MSG) D Chicken essence seasoning E Oyster sauce F Soy sauce G Sweet Soy Sauce H None I Don’t know

8. Have you ever heard of the nutrition facts label on food products?

A Yes B No (Skip to the Salt-related Attitude section)

9. In the nutrition facts label, which item represents the salt content of a food product?

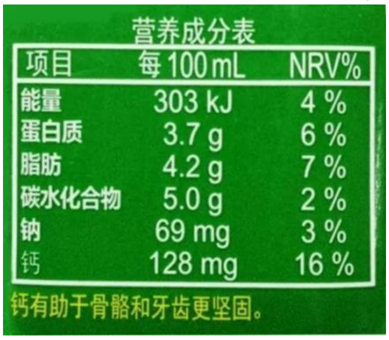

营养成分表

项目	每100mL	NRV%
能量	303 kJ	4 %
蛋白质	3.7 g	6 %
脂肪	4.2 g	7 %
碳水化合物	5.0 g	2 %
钠	69 mg	3 %
钙	128 mg	16 %

钙有助于骨骼和牙齿更坚固。

A Energy B Protein C Fat D Carbohydrates E Sodium F Calcium G Don’t know

III. Salt-related Attitudes

1. Do the following statements describe your situation? (For each statement, please select the answer that best reflects your actual situation and mark “√” in the corresponding box.)

Items	Strongly agree	Agree	Neutral	disagree	Strongly disagree
1 I think that it is necessary to promote salt reduction among children aged 3-6 years.					
2 I would like to learn more about health knowledge related to salt intake.					
3 I would like to learn practical tips for reducing salt intake.					
4 I am willing to use salt-control tools during family cooking.					
5 I am willing to reduce my child’s intake of high-salt snacks.					
6 I am willing to use less salt when preparing meals for my child.					

2. What are the potential barriers to reducing salt when preparing meals for your child? (Multiple answers are allowed.)

A My child may not like the food if less salt is added.

B I have become accustomed to the current amount of salt used in cooking, and it is difficult to change.

C It is inconvenient or troublesome.

D I do not know how to control my child’s salt intake.

E Other reasons: _______

F No barriers.

IV. Salt-related Practices

(Ⅰ) Salt Use During Household Cooking

1. When preparing pre-packaged foods (e.g., ready-to-eat meals, instant noodles), how do you usually use the seasoning packets?

A Use only a small portion of the seasoning packet (approximately one-third).

B Use most of the seasoning packet (approximately two-thirds).

C Use the entire seasoning packet.

D Use the entire seasoning packet and add additional seasonings.

E I never consume pre-packaged foods such as ready-to-eat meals or instant noodles.
2. Do you currently use salt-control spoons or salt-control containers?
A I have been using them continuously.
B I have used them before but no longer use them.
C I have never used them.
D I have never heard of salt-control spoons and/or salt-control containers.
3. What type of salt has your household mainly used for cooking during the past month?
A Regular salt B Low-sodium salt C Iodized salt
4. When cooking at home, do you have the following habits? (For each item, please select the answer that best reflects your actual situation and mark "√" in the corresponding box.)

Items	Never	Occasionally	Sometimes	Often	Always
1 Add salt at the end of cooking.					
2 After adding seasonings such as dark soy sauce, soy sauce paste, fermented bean paste, or oyster sauce to dishes, consciously reduce the amount of salt added.					
3 Use natural herbs and spices (e.g., scallions, ginger, garlic, chili, and pepper) to enhance food flavor and reduce the amount of salt used during cooking.					

(Ⅱ) Dietary Salt Intake Behaviors
5. How are meals usually served in the kindergarten cafeteria where your child attends?
A Food and staple foods are served separately.
B Food and staple foods are mixed together.
C Both situations occur.
D I Don't know.
6. How often does your family use dipping sauces with meals in a typical week?
A Almost every day B 3-5 days per week C 1-2 days per week D Never (Skip to Question 19)
7. What salty seasonings are usually added to the dipping sauces used by your family? (Multiple answers are allowed.)
A Monosodium glutamate (MSG) / chicken essence B Salt C Soy sauce D Oyster sauce
E Fermented bean curd
F Sauces (e.g., spicy sauce, peanut sauce, chopped chili sauce)
8. During the past six months, how often did the following dietary behaviors occur? (For each item, please select the answer that best reflects your actual situation and mark "√" in the corresponding box.)

Items	Never	Occasionally	Sometimes	Often	Always
1 When eating noodle dishes with soup, do you drink the soup in the bowl?					
2 When your child eats noodle dishes with soup, does your child drink the soup in the bowl?					
3 How often do you eat at restaurants or order takeout?					
4 How often does your child eat at restaurants or have takeout food?					
5 When eating at restaurants or ordering takeout, do you actively request less salt to be added?					
6 Do you rinse your child's food with water separately because you are concerned that the food					

may be too salty?					

(Ⅲ) Hidden Salt Intake

9. When choosing snacks for your child, do you compare the salt content shown on nutrition labels?

A Never B Occasionally C Sometimes D Often E Always

10. During the past month, how often did you or your child consume the following foods? (For each item, please select the answer that best reflects the actual situation and mark "√" in the corresponding box.)

Items	Almost every day	3-5 days per week	1-2 days per week	No more than once a week
1 Your consumption of pickled foods.				
2 Your child's consumption of pickled foods.				
3 Your consumption frequency of salty snacks, including rice crackers, potato chips, dried meat, dried tofu, ham sausages, preserved plums, and salty biscuits.				
4 Your child's consumption frequency of salty snacks, including rice crackers, potato chips, dried meat, dried tofu, ham sausages, preserved plums, and salty biscuits.				

This questionnaire ends here. Thank you!

Scoring Criteria for the Questionnaire on Salt Intake Status and Salt-Reduction Knowledge, Attitudes, and Practices among Caregivers of Preschool Children

<table>
<tr><th>Maximum score</th><th>Source of items</th><th colspan="5">Item options and scoring criteria</th></tr>
<tr><td colspan="7">Knowledge of Salt and Health (K)</td></tr>
<tr><td>10 points</td><td colspan="6">Included five single-choice items and one multiple-choice item. Correct answers were assigned 1 point, whereas “unclear” or “unknown” responses were assigned 0 points. Higher scores indicated better knowledge.</td></tr>
<tr><td rowspan="8">Seven items were scored, with a maximum possible score of 10 points.</td><td>2.2 Do you think that eating less salt can cause people to feel weak?</td><td colspan="5">B No</td></tr>
<tr><td>2.3 What is the maximum recommended daily salt intake for preschool children?</td><td colspan="5">B 3 g</td></tr>
<tr><td>2.4 What is the maximum recommended daily salt intake for adults?</td><td colspan="5">D 5 g</td></tr>
<tr><td>2.5 Excessive salt intake over a long period may increase the risk of which diseases? (Multiple choices allowed)</td><td colspan="5">six options (A Hypertension B Gastric cancer C Obesity D Osteoporosis E Stroke F Kidney disease) were correct. Participants received 1 point for selecting one correct option, 2 points for selecting 2-3 correct options, 3 points for selecting 4-5 correct options, and 4 points for selecting all six correct options. Selecting “G Don’t know” received 0 points.</td></tr>
<tr><td>2.6 Which of the following snacks do you think do not contain salt? (Multiple choices allowed)</td><td colspan="5">G None</td></tr>
<tr><td>2.7 Which of the following foods and seasonings do you think do not contain salt? (Multiple choices allowed)</td><td colspan="5">H None</td></tr>
<tr><td>2.8 Have you ever heard of the nutrition facts label on food products?</td><td colspan="5">A Yes</td></tr>
<tr><td>2.9 In the nutrition facts label, which item represents the salt content of a food product?</td><td colspan="5">E Sodium
selecting an incorrect option or choosing “unknown” was assigned −1 point, whereas selecting the correct option or not selecting this option was assigned 0 points. The score of this item was combined with the score of Question 2.8 to calculate the subtotal score for food nutrition label knowledge.</td></tr>
<tr><td colspan="7">Salt-related Attitudes (A)</td></tr>
<tr><td>24 points</td><td colspan="6">A five-point Likert scale was used for scoring. The assigned scores varied according to the wording and response options of each item. Higher scores indicated more positive attitudes toward salt reduction.</td></tr>
<tr><td>Six items were</td><td>Source of items</td><td>Strongly agree</td><td>Agree</td><td>Neutral</td><td>disagree</td><td>Strongly disagree</td></tr>
</table>

scored, with each item contributing 4 points and a maximum possible score of 24 points.	3.1.1 I think that it is necessary to promote salt reduction among children aged 3-6 years.	4	3	2	1	0
	3.1.2 I would like to learn more about health knowledge related to salt intake.	4	3	2	1	0
	3.1.3 I would like to learn practical tips for reducing salt intake.	4	3	2	1	0
	3.1.4 I am willing to use salt-control tools during family cooking.	4	3	2	1	0
	3.1.5 I am willing to reduce my child’s intake of high-salt snacks.	4	3	2	1	0
	3.1.6 I am willing to use less salt when preparing meals for my child.	4	3	2	1	0
Salt-related Practices (P)						
60 points	A five-point Likert scale was used for scoring. The assigned scores varied according to the wording and response options of each item. Higher scores indicated healthier salt-reduction Practices.					
Seventeen items were scored, with a maximum possible score of 60 points.	4.1 When preparing pre-packaged foods (e.g., ready-to-eat meals, instant noodles), how do you usually use the seasoning packets?	A Use only a small portion of the seasoning packet (approximately one-third)---3 points<br>B Use most of the seasoning packet (approximately two-thirds)---2 points<br>C Use the entire seasoning packet---1 point<br>D Use the entire seasoning packet and add additional seasonings---0 points<br>E I never consume pre-packaged foods such as ready-to-eat meals or instant noodles---4 points				
	4.2 Do you currently use salt-control spoons or salt-control containers?	A I have been using them continuously---2 points<br>B I have used them before but no longer use them---1 point<br>C I have never used them---0 points<br>D I have never heard of salt-control spoons and/or salt-control containers---0 points				
	Source of items	Never	Occasionally	Sometimes	Often	Always
	4.4.1 Add salt at the end of cooking.	0	1	2	3	4
	4.4.2 After adding seasonings such as dark soy sauce, soy sauce paste, fermented bean paste, or oyster sauce to dishes, consciously reduce the amount of salt added.	0	1	2	3	4
	4.4.3 Use natural herbs and spices (e.g., scallions, ginger, garlic, chili, and pepper) to enhance food flavor and reduce the amount of	0	1	2	3	4

	salt used during cooking.					
	4.6 How often does your family use dipping sauces with meals in a typical week?	A Almost every day---0 points<br>B 3-5 days per week---1 point<br>C 1-2 days per week---2 points<br>D Never (Skip to Question 19)---2 points				
	4.8.1 When eating noodle dishes with soup, do you drink the soup in the bowl?	4	3	2	1	0
	4.8.2 When your child eats noodle dishes with soup, does your child drink the soup in the bowl?	4	3	2	1	0
	4.8.3 How often do you eat at restaurants or order takeout?	4	3	2	1	0
	4.8.4 How often does your child eat at restaurants or have takeout food?	4	3	2	1	0
	4.8.5 When eating at restaurants or ordering takeout, do you actively request less salt to be added?	0	1	2	3	4
	4.8.6 Do you rinse your child's food with water separately because you are concerned that the food may be too salty?	0	1	2	3	4
	4.9 When choosing snacks for your child, do you compare the salt content shown on nutrition labels?	0	1	2	3	4

	Source of items	Almost every day	3-5 days per week	1-2 days per week	No more than once a week
	4.10.1 Your consumption of pickled foods.	0	1	2	3
	4.10.2 Your child's consumption of pickled foods.	0	1	2	3
	4.10.3 Your consumption frequency of salty snacks, including rice crackers, potato chips, dried meat, dried tofu, ham sausages, preserved plums, and salty biscuits.	0	1	2	3
	4.10.4 Your child's consumption frequency of salty snacks, including rice crackers, potato chips, dried meat, dried tofu, ham sausages, preserved plums, and salty biscuits.	0	1	2	3
