# Supplementary material for: Effects of Salt-Reduction Health Education Short Videos on Salt-Related Knowledge, Attitudes, and Practices (KAP) Among Caregivers of Preschool Children
Source: Nutrients. 2026 Oct 26;18(21):3525. doi: 10.3390/nu18213525 (PMC13764901; doi:10.3390/nu18213525)
Supplement: Supplementary file 1 [file nutrients-18-03525-s001.zip › Suppl. Mat. File S2 - Comparison of core health education content between print media materials and salt-reduction health education short videos.pdf]

Supplementary Materials

Effects of Salt-Reduction Health Education Short Videos on Salt-Related Knowledge, Attitudes, and Practices (KAP) Among Caregivers of Preschool Children

File S2. Comparison of core health education content between print media materials and salt-reduction health education short videos.

Figure	Salt-Reduction Health Education Short Videos	Print Media Materials
A	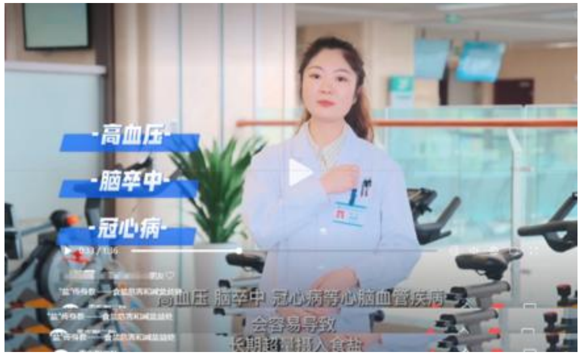	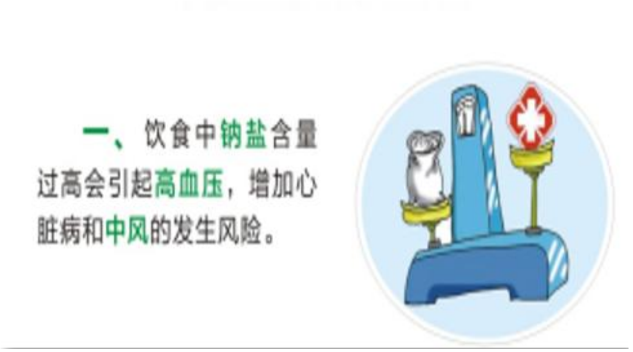
	The short videos introduce the health risks associated with excessive salt intake.	The print media materials present the health risks associated with high salt intake.
B	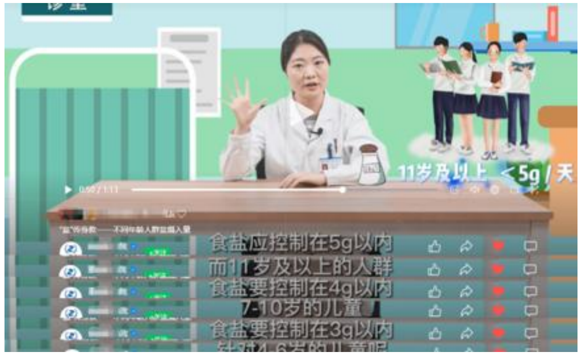	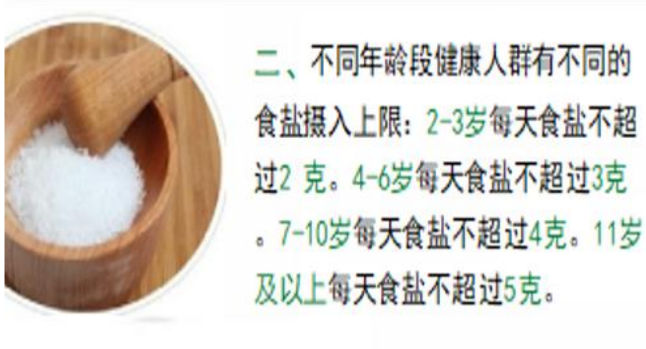
	The short videos introduce the recommended salt intake for different age groups.	The print media materials present the recommended upper limits of salt intake for different age groups from age 2 to adulthood.

<table>
<tr><td>C</td><td>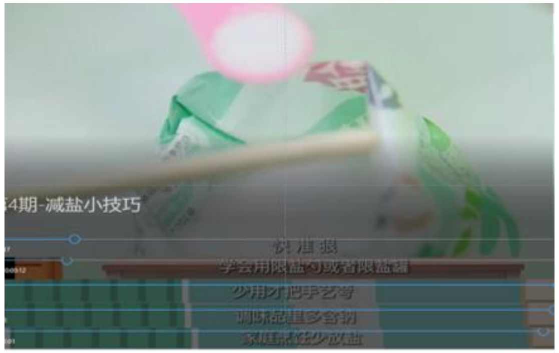</td><td>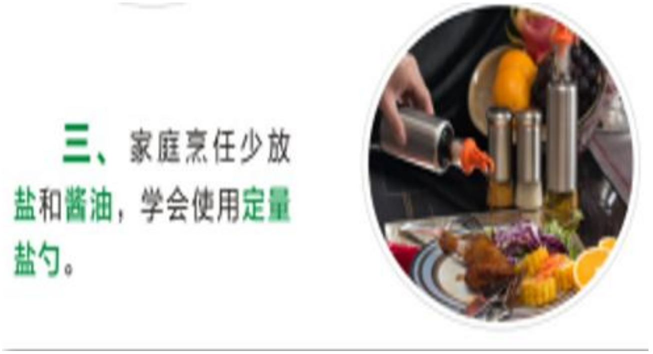</td></tr>
<tr><td></td><td>The short videos introduce ways to reduce salt use in home cooking and use salt-control tools.</td><td>The print media materials advocate using less salt in home cooking and using a salt-measuring spoon.</td></tr>
<tr><td>D</td><td>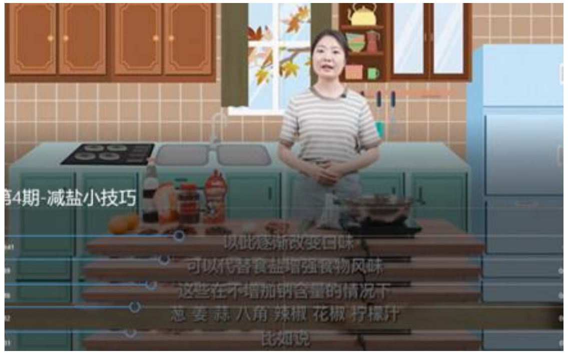</td><td>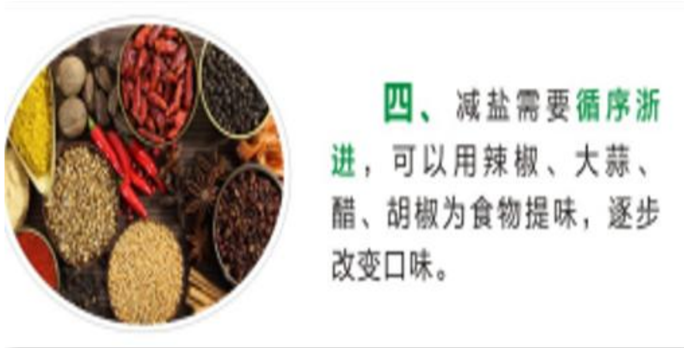</td></tr>
<tr><td></td><td>The short videos introduce the use of flavor-enhancing ingredients to improve food flavor and gradually change taste preferences</td><td>The print media materials advocate gradual salt reduction and the use of flavor-enhancing ingredients to improve food flavor.</td></tr>
<tr><td>E</td><td>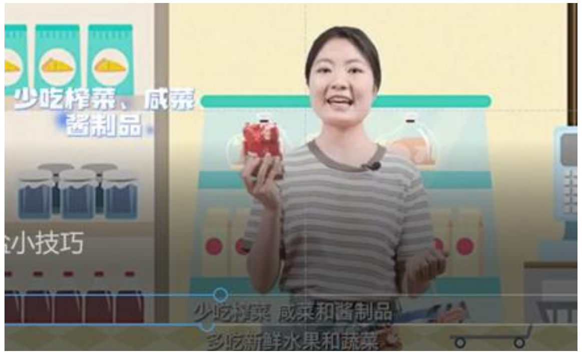</td><td>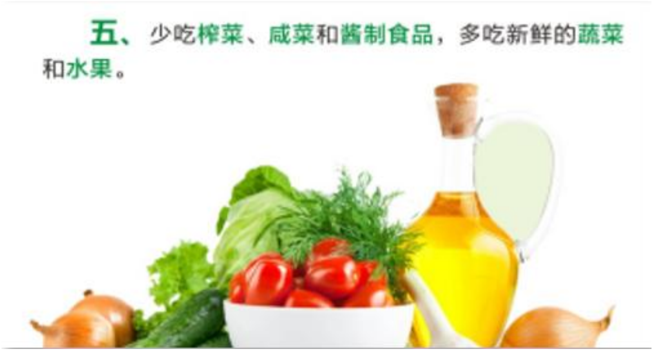</td></tr>
</table>

<table>
<tr><td></td><td>The short videos advocate eating more fresh fruits and vegetables and less pickled and preserved foods.</td><td>The print media materials advocate eating less pickled and preserved foods, and more fresh fruits and vegetables.</td></tr>
<tr><td rowspan="2">F</td><td>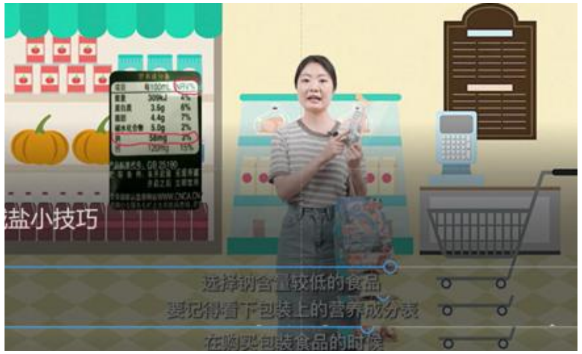
</td><td>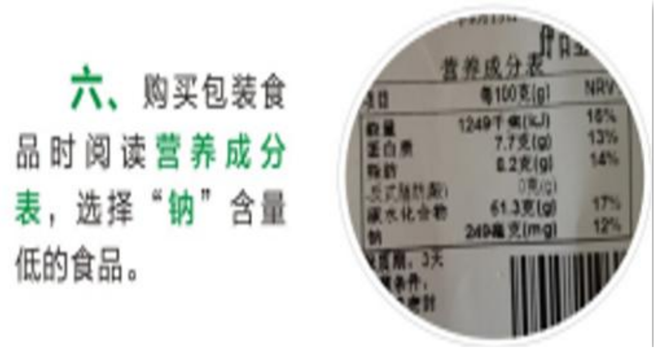
</td></tr>
<tr><td>The short videos demonstrate how to read nutrition labels and select low-sodium foods.</td><td>The print media materials illustrate how to select low-sodium foods based on nutrition labels.</td></tr>
<tr><td rowspan="2">G</td><td>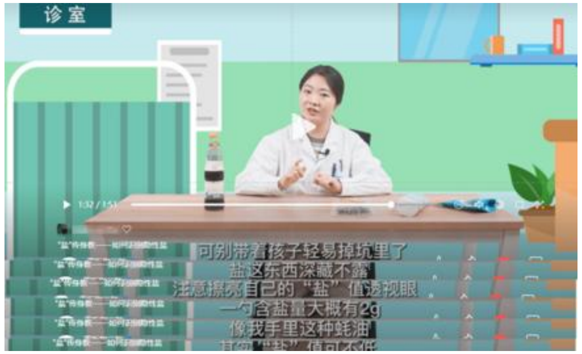
</td><td>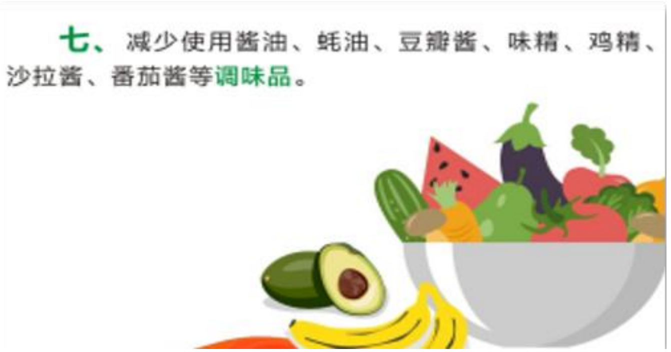
</td></tr>
<tr><td>The short videos present the salt content of selected condiments and advise reducing their use.</td><td>The print media materials advocate reducing the use of condiments.</td></tr>
</table>

<table>
<tr><td>H</td><td>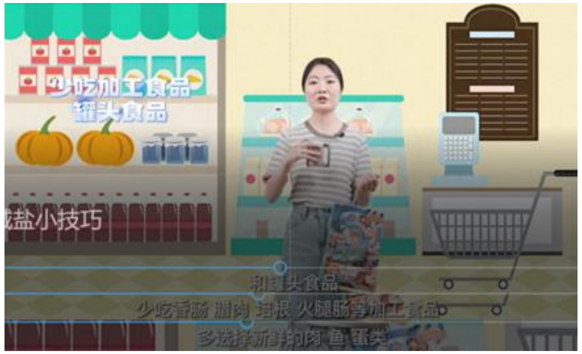
</td><td>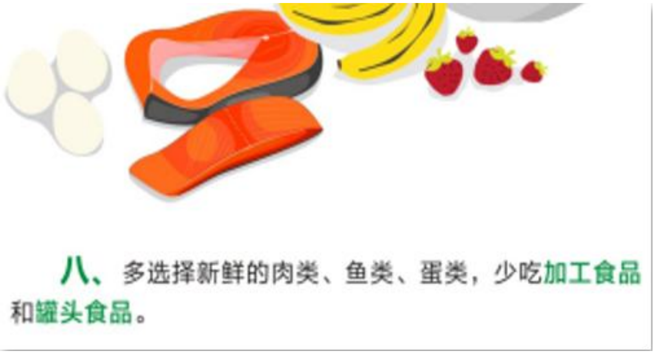
</td></tr>
<tr><td></td><td>The short videos advocate choosing more fresh fish, meat, and eggs and reducing the intake of processed foods.</td><td>The print media materials advocate choosing more fresh fish, meat, and eggs and reducing the intake of processed foods.</td></tr>
<tr><td>I</td><td>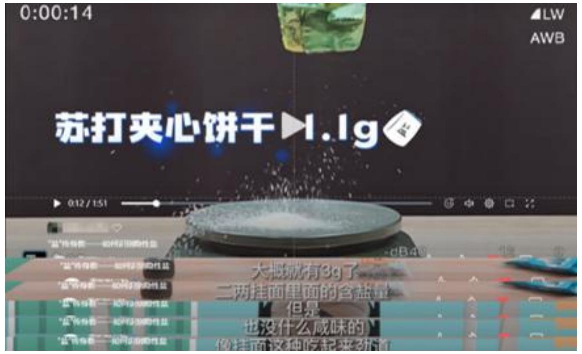
</td><td>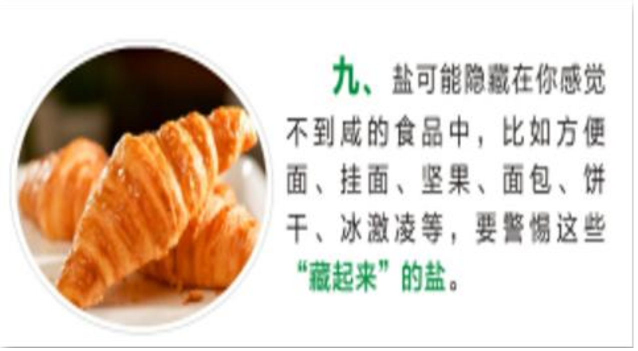
</td></tr>
<tr><td></td><td>The short videos introduce the presence of “hidden salt” in various foods.</td><td>The print media materials highlight “hidden salt” in foods.</td></tr>
<tr><td>J</td><td>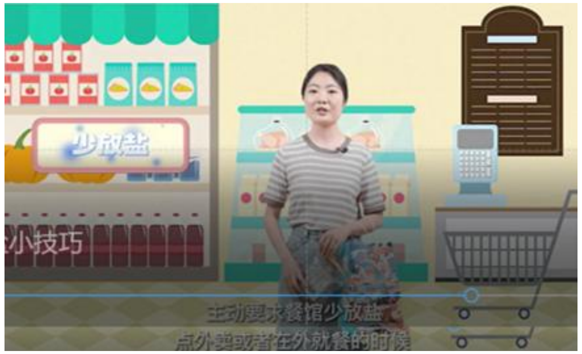
</td><td>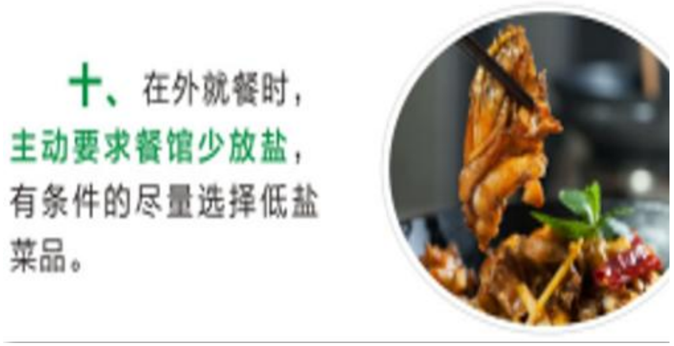
</td></tr>
<tr><td></td><td>The short videos remind viewers to request less salt when eating out.</td><td>The print media materials advocate requesting less salt when eating out.</td></tr>
</table>
